# Large Language Model-Generated Differential Diagnoses in Radiology Education: Comparison with a Standard Casebook

**DOI:** 10.3390/diagnostics16132009

**Published:** 2026-06-27

**Authors:** Pauline Chapellier, Jacopo Ferrari, Thomas Saliba, Patrick Jeltsch, Mustafa Mohamed, Sofyan Jankovski, Gorun Ilanjian, Marta Epis, Virginia Pansini, Federica Bragaglia, Alessandro Agostinelli, Krismalyn Caringal, Lachezar Lalov, David C. Rotzinger, Guillaume Fahrni

**Affiliations:** 1 Department of Diagnostic and Interventional Radiology, Lausanne University Hospital, University of Lausanne, 1011 Lausanne, Switzerland; 2 ASST Grande Ospedale Metropolitano Niguarda, Università Degli Studi di Milano, 20162 Milano, Italy; 3 Faculty of Medicine, Université Libre de Bruxelles, 1050 Brussels, Belgium

**Keywords:** radiology education, large language models, differential diagnosis, ChatGPT, medical training

## Abstract

**Background/Objectives**: Large language models (LLMs) are increasingly explored for radiology education, but their role in differential diagnosis learning remains under-investigated. This study evaluates the perceived usefulness of LLM-generated differential diagnoses compared with a standard radiology casebook. **Methods**: In this multi-center study, radiology trainees at junior (years 1–2) and advanced (years 3–5) levels evaluated 225 cases from a gold-standard casebook spanning nine subspecialties. Participants ranked the usefulness of their personal clinical experience, the casebook, and LLM teaching, and rated the LLM output using a five-point Likert scale across Clarity, Trust, Differential Usefulness, and Diagnostic Usefulness. **Results**: Thirteen trainees (4 junior, 9 advanced) completed 2425 evaluations. Overall, the casebook was rated most useful (mean rank 1.7 ± 0.2), followed by LLM teaching (1.8 ± 0.3) and personal experience (2.4 ± 0.2; *p* = 0.023), with no significant difference between LLM and Textbook (*p* = 0.438). Junior trainees favored LLM teaching more than advanced trainees (first-rank 66.6% vs. 22.1%; *p* = 0.037). Across subspecialties, the casebook consistently ranked highest, with LLM slightly lower and experience lowest. LLM teaching received high ratings for Clarity (4.4 ± 0.3), Trust (4.3 ± 0.3), Differential Usefulness (4.3 ± 0.4), and Diagnostic Usefulness (4.2 ± 0.4), with no statistically significant difference between domains (*p* = 0.149). **Conclusions**: LLM-generated differential diagnoses are clear, trustworthy, and perceived as highly useful for education, nearing the perceived value of a standard casebook, especially for junior trainees. While textbooks remain essential, LLMs hold promise as supplementary tools, but caution is needed due to potential inaccuracies and their inability to replicate image-based teaching.

## 1. Introduction

Over the past decades, artificial intelligence (AI) has become a major research focus in the medical field. The advent of large language models (LLMs) has led to a substantial shift in AI applications across medical disciplines. These models undergo pretraining on extensive datasets, enabling them to both generate and comprehend natural language text [1,2].

Several studies have demonstrated the utility of LLMs in diagnostic and screening workflows by collecting medical information and integrating statistical data and disease incidence [3,4,5]. LLMs also play an increasing role in patient care by optimizing surgical planning [6], improving workflow management, supporting clinical decision-making, and even suggesting the most appropriate treatment for individual patients [7,8,9].

The use of LLMs in radiology remains limited but holds considerable potential. These models could assist in selecting the most appropriate imaging modality and protocol based on the clinical context, or even automate this process [10,11]. Most existing studies in radiology AI focus on image assessment, diagnosis and screening, and making standardized reports, with the aim of improving radiologist sensitivity and increasing daily productivity [12,13,14].

ChatGPT (OpenAI, GPT-3.5, San Francisco, CA, USA) in November 2022 marked a significant turning point in the application of medical LLMs, especially for medical students [15]. This tool has demonstrated promising capabilities in supporting medical students by facilitating knowledge acquisition, improving clinical reasoning, and assisting with exam preparation through interactive, case-based learning [16,17]. Indeed, the broader implementation of ChatGPT across healthcare has underscored major opportunities for knowledge retrieval and clinical support, alongside persistent challenges regarding factual reliability [18]. In the field of radiology, ChatGPT and other large language models, such as DeepSeek (DeepSeek AI, Hangzhou, China), are beginning to be investigated and have shown potential for radiology education [19,20]. Parallel efforts have focused on benchmarking LLM diagnostic accuracy against radiologists across subspecialties, including cardiac and pediatric neuroradiology cases, further delineating the capabilities and limitations of these models in clinically relevant settings [21,22]. However, these performance-oriented studies differ fundamentally from an educational perspective, as they assess LLM accuracy rather than the value of LLM-generated content as a structured learning tool for trainees.

Radiology training and education relies particularly on a comprehensive understanding of differential diagnoses for a given imaging finding, which must then be prioritized according to clinical information. This pedagogical approach reflects the reality of clinical radiology, where most imaging findings are non-specific and rarely correspond to a single definitive diagnosis. Such educational approaches are traditionally based on the study of textbooks and collections of clinical cases (so-called casebooks) [23]. These specialized casebooks represent heavily peer-reviewed distillations of knowledge authored by top-tier international subspecialists. ChatGPT holds significant potential for specialty-specific training; however, its value in radiology education and differential diagnosis generation remains insufficiently explored [12,24]. Comparing LLM outputs directly against an established casebook is pedagogically meaningful because it evaluates whether interactive, AI-driven text generation can match or supplement the structured active recall traditionally provided by gold-standard print literature.

Unlike most existing studies, which evaluate LLMs primarily for diagnostic accuracy, report generation, or radiologist-level benchmarking, the perceived educational value and usefulness of LLM-generated differential diagnoses as a structured learning tool remain largely unexplored. Radiology differential diagnosis training is uniquely suited to LLM support because navigating non-specific imaging features requires synthesizing dense text-based clinical data, demographic factors, and probabilistic reasoning, areas where semantic synthesis engines excel.

Thus, the aim of this study was to compare the perceived usefulness of differential diagnoses generated by ChatGPT-4o with those provided in the text-based differential diagnosis teaching sections of a standard radiology casebook, using predefined imaging themes (key imaging findings) as a common starting point for junior and advanced radiology trainees. We hypothesized that while LLM-generated differential diagnoses may offer promising perceived educational support, they also present limitations and caveats that are important to recognize given their likely widespread adoption in radiology training.

## 2. Materials and Methods

### 2.1. Study Design

This comparative within-subjects educational study was conducted between March and October 2025 across three academic centers: Lausanne University Hospital and the University of Lausanne (Switzerland), Università degli Studi di Milano—ASST Grande Ospedale Metropolitano Niguarda (Italy), and the Faculty of Medicine of Université Libre de Bruxelles (Belgium). The study aimed to compare differential diagnosis teaching derived from a standard radiology casebook with teaching generated by an LLM.

**Figure 1:**
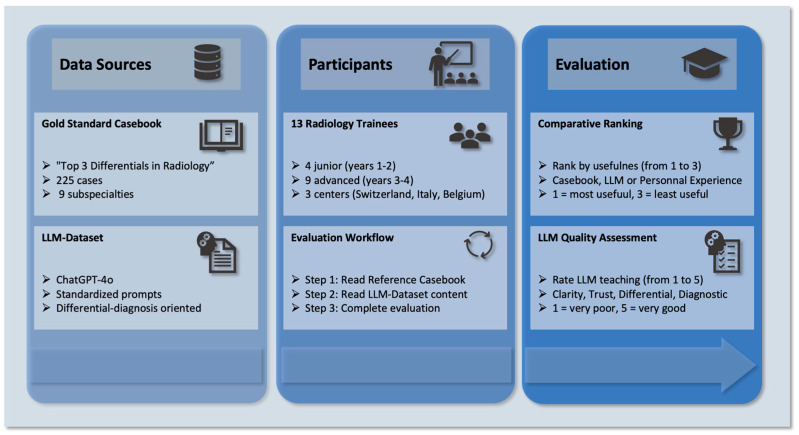
Study Workflow and Evaluation Protocol. Overview of the study design showing the three main components: data sources (reference casebook and LLM-generated dataset), participants and evaluation workflow, and assessment criteria (comparative ranking and LLM quality evaluation).

Radiology trainees at different stages of postgraduate training were recruited, including junior residents (years 1–2) and advanced residents (years 3–5). Recruitment was conducted informally through collegial networks. Participation was voluntary, and no formal inclusion or exclusion criteria were applied. Participants were informed that involvement in the study could provide an opportunity to deepen their radiological knowledge and gain exposure to LLM-based educational tools. The overall study workflow is illustrated in Figure 1.

### 2.2. Gold Standard Casebook

**Figure 2:**
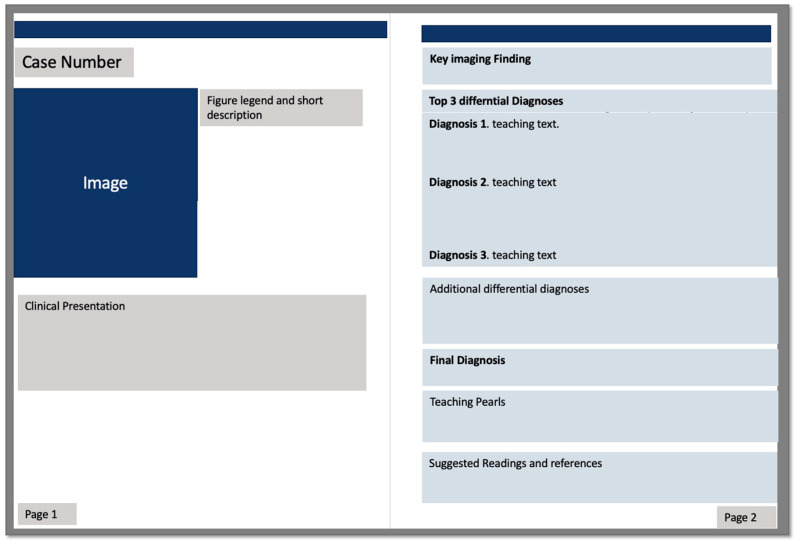
Schematic Representation of Casebook Case Structure. Illustration of the typical two-page layout from the reference casebook. The left page presents the clinical scenario and radiological images, while the right page provides the Key Imaging Findings and differential diagnosis teaching. This structure is the same for each case. This represents the format of the cases accessed and reviewed by participants during the study.

The reference standard for differential diagnosis teaching was the textbook “Top 3 Differentials in Radiology: A Case Review (O’Brien WT, ed. New York: Thieme Medical Publishers; 2010)” [25]. This casebook presents clinically relevant educational radiology cases organized around key imaging findings. Each case follows a structured format, beginning with a brief clinical introduction and the presentation of radiologic images depicting a key lesion. At this stage, the underlying thematic imaging finding (e.g., “Solitary Pulmonary Nodule”) is not disclosed to the reader, who is expected to identify the imaging pattern, formulate a differential diagnosis, and propose a final diagnosis. The case concludes with the formal identification of the thematic lesion, the top three differential diagnoses, and the confirmed final diagnosis. A schematic template illustrating the general structure of a typical case is provided in Figure 2.

The casebook covers a broad range of radiology subspecialties, including Chest and Cardiac, Gastrointestinal, Genitourinary, Musculoskeletal, Head and Neck, Brain and Spine, Pediatric, Ultrasound, Fetal, Vascular and Interventional, Nuclear Medicine, Breast, and Roentgen Classics. Each subspecialty section comprises 25 cases. For this study, all sections were included except Nuclear Medicine, Ultrasound Imaging, Fetal Imaging, and Roentgen Classics. These categories were excluded either because they were not consistently represented in the participating centers’ curricula or because they extended beyond the scope of classical imaging-based differential diagnosis teaching targeted in this evaluation. Furthermore, only the textual differential diagnosis teaching component associated with each thematic lesion was evaluated. The image-based quiz component of the casebook was not included in the comparison, as current LLM systems do not reliably generate coherent and pedagogically valid radiologic images.

### 2.3. LLM-Dataset Generation

The LLM-generated educational dataset was produced using ChatGPT-4o (OpenAI, web-based interface, March 2025 version). To minimize personalization effects, a newly created user account was used, and conversation memory was disabled. For each individual thematic topic, a new chat session was initiated to avoid contextual carryover between cases and to prevent progressive expansion of the context window.

**Figure 3:**
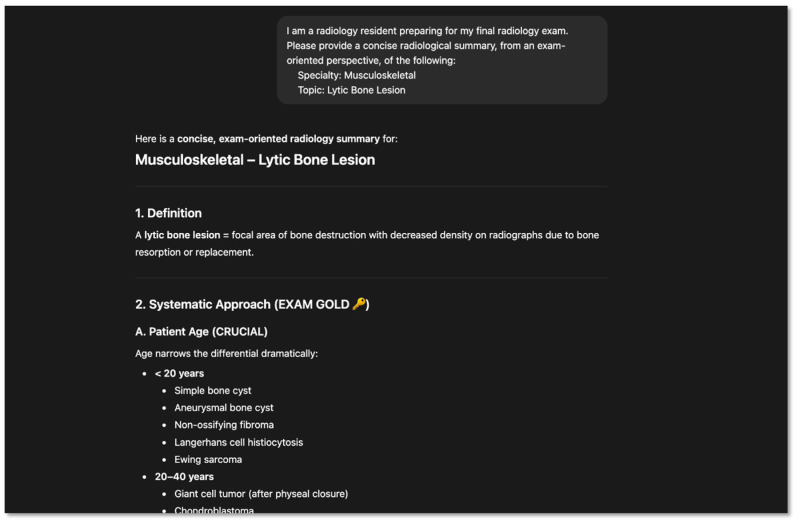
Example of Structured Prompt and LLM Response. Illustration of the standardized prompt template used to generate the LLM-dataset, showing sections for Topic, Specialty, and Key Imaging Findings. The prompt is followed by the LLM response output (partially visible), demonstrating the typical format of LLM-generated differential diagnosis teaching content.

For every case topic, a standardized structured prompt was employed. The only variables modified across prompts were the thematic subject corresponding to the “Key Imaging Findings” section of the reference casebook (for example, “Cavitary Pulmonary Mass”) and the name of the subspecialty (for example, “Head and Neck”). The prompt was developed through multiple internal iterations of prompt engineering until a robust and reproducible format was achieved, generating responses resembling structured differential diagnosis teaching. An example of the prompt and a corresponding excerpt of the output is provided in Figure 3.

It is important to note that, while all prompts and generation settings were standardized, the use of a commercial web-based interface imposes inherent limitations on reproducibility. Specifically, internal model parameters such as temperature, system-level configurations, and potential backend updates are not accessible to end users and could therefore not be controlled or reported. As a consequence, exact replication of identical outputs cannot be guaranteed, even when using the same prompt, model, and interface conditions.

All prompts and responses were generated exclusively in English. The outputs were copied verbatim into Word documents organized by subspecialty without any content editing. Formatting elements such as bold text, bullet points, and paragraph structures were preserved. Conversational closing phrases occasionally produced by the model (e.g., invitations for further clarification) were intentionally retained to reflect authentic LLM output. The complete dataset, entitled RAD-CaseBookLLM-08, which includes a dedicated documentation file specifying the exact prompt template, model specifications, generation settings, and reproducibility details, is freely available as open-access data on Zenodo [26,27] under a CC0 1.0 Universal license (https://doi.org/10.5281/zenodo.18667708).

### 2.4. LLM-Dataset Evaluation

Each participant was provided with access to both the reference casebook and the complete LLM-generated dataset. For each case, participants were instructed to first read the corresponding case in the casebook in order to familiarize themselves with the gold standard differential diagnosis teaching for the topic. This sequential design was deliberately chosen to provide non-expert trainees with a standardized baseline and mitigate automation bias, although it inherently introduces a priming effect that may not fully replicate an independent, unassisted clinical learning setting. Participants subsequently reviewed the LLM-generated teaching for the same topic to judge its quality relative to the gold standard casebook and to assess how both forms of teaching related to their previous clinical experience. Participants were free to determine the total number of cases they wished to evaluate. A minimum of two full sections (at least 50 cases) was required. No time limit was imposed, as the study aimed to simulate a realistic self-directed revision setting rather than a time-constrained clinical environment.

For each evaluated case, participants performed two qualitative assessments. First, they ranked the relative usefulness of three sources, namely the gold standard casebook, the LLM-generated dataset, and their own personal clinical knowledge, using an ordinal ranking (1 = most useful; 3 = least useful). This ranking aimed to determine whether the LLM-generated teaching was perceived as more or less useful than the textbook or than personal experience, and whether educational materials were perceived differently depending on the level of training.

Second, participants evaluated the LLM-generated content using a five-point Likert scale across four predefined domains: Clarity (clarity of the text), Trust (perceived trustworthiness), Differential Usefulness (usefulness for understanding the differential diagnosis), and Diagnostic Usefulness (usefulness for understanding the final diagnosis). Each domain was rated on a scale from 1 to 5, where 1 corresponded to “very poor,” 2 to “poor,” 3 to “average,” 4 to “good,” and 5 to “very good.” Each case was evaluated once, and no repeated assessments were performed. Given that participants were radiology trainees rather than experts, they lacked an independent reference to objectively rate the casebook. The reference casebook was therefore treated as the fixed benchmark and assigned maximal scores (5/5/5/5) by design, as the study objective was to assess how closely LLM-generated content approached this established reference rather than to evaluate the casebook’s intrinsic quality. Participants therefore qualitatively assessed the LLM-generated content relative to this fixed benchmark.

### 2.5. Statistical Analysis

All statistical analyses were performed using RStudio (Version 2026.01.0+392; RStudio, PBC, Boston, MA, USA). Descriptive statistics were reported as mean ± standard deviation or median with interquartile range (IQR), depending on the distribution of the data.

For the ranking evaluation, to account for the clustering of repeated evaluations within participants, inferential analyses were conducted on participant-level aggregated data. For each participant, mean ranks were computed per modality across all evaluated cases prior to statistical testing. A Friedman test was applied to assess whether mean ranks differed significantly across the three modalities. When the Friedman test was significant, post hoc pairwise comparisons were performed using Wilcoxon signed-rank tests with Bonferroni correction for multiple comparisons. To examine whether perceived usefulness differed between junior and advanced radiologists, mean ranks per participant for each modality were compared between groups using the Mann–Whitney U test.

For the qualitative evaluation of the LLM-dataset, participant-level mean Likert scores were similarly computed per domain prior to inferential testing. Differences between domains were analyzed using a Friedman test with post hoc Wilcoxon signed-rank pairwise comparisons and Bonferroni correction when appropriate. Differences between junior and advanced radiologists were assessed using the Mann–Whitney U test. Similarly, to explore domain differences within each radiology subspecialty, a Friedman test was applied per specialty, with post hoc Wilcoxon signed-rank tests as appropriate. A *p*-value < 0.05 was considered statistically significant.

## 3. Results

### 3.1. Characteristics of Participants and Evaluations

A total of 13 radiology trainees from three European academic centers (Switzerland, Italy and Belgium) participated in the study between March and October 2025. The cohort included 4 junior trainees (postgraduate years 1–2) and 9 advanced trainees (postgraduate years 3–5). Participants collectively evaluated a total of 2425 case-based questions from the RAD-CaseBookLLM-08 dataset, using the gold standard casebook as a reference.

All trainee radiologists completed the full set of 225 questions. Among advanced trainees, the number of evaluated questions was more variable, ranging from 50 to 225, with a mean of 169.4 ± 68.2, corresponding to an average completion rate of 75% for this group.

The distribution of evaluated cases spanned all nine selected radiology subspecialties, with the highest coverage observed in Neuroradiology (92.3% of all possible questions) and the lowest in Breast Imaging (61.5%). Detailed participant characteristics and the breakdown of evaluated cases by subspecialty and training level are presented in Table 1.

### 3.2. Ranking Evaluation

**Figure 4:**
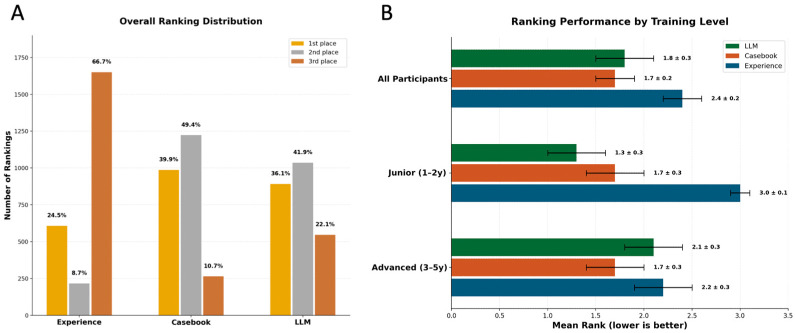
Comparative Ranking Performance Across Sources and Training Levels. (**A**) Overall ranking distribution showing the frequency of first-, second-, and third-place rankings for Experience, Casebook, and LLM across all evaluations. Percentages indicate the proportion within each source. (**B**) Mean ranking performance (±standard deviation) stratified by participant training level (overall, junior residents [1–2 years], advanced residents [3–5 years]). Lower mean ranks indicate better performance (1 = best, 3 = worst). Error bars represent standard deviation.

**Figure 5:**
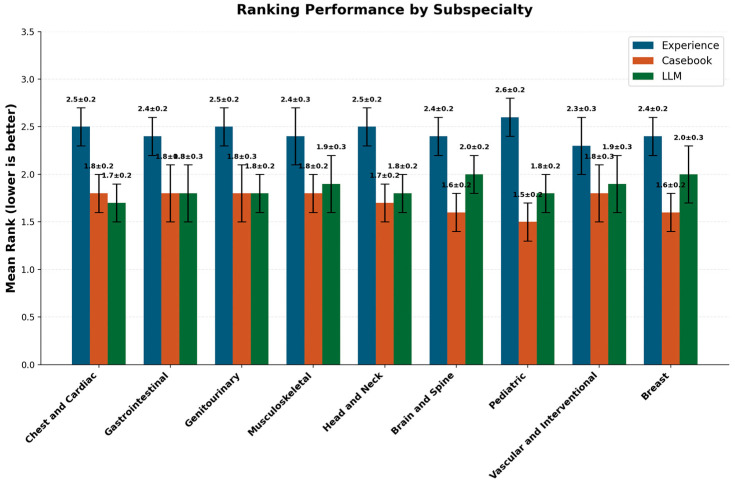
Mean Ranking Performance Across Radiology Subspecialties. Mean ranking performance (±standard deviation) across nine radiology subspecialties. Lower mean ranks indicate better performance (1 = best, 3 = worst). Error bars represent standard deviation.

All results for ranking evaluation are summarized in Table 2, Figure 4 and Figure 5. Overall, when considering all participants, the reference casebook was perceived as the most useful source for learning radiology differential diagnoses, followed closely by the LLM-generated teaching, while participants’ personal clinical experience was generally ranked as least useful. The mean ranks across all cases reflected this pattern, with the casebook achieving a mean rank of 1.7 ± 0.2, the LLM-dataset 1.8 ± 0.3, and personal experience 2.4 ± 0.2. In terms of ordinal rankings, the casebook was assigned first rank in 40% of cases, the LLM in 36%, and personal experience in only 25%. A Friedman test applied to participant-level aggregated data confirmed statistically significant differences between the three sources (χ^2^ = 7.54, df = 2, *p* = 0.023, Kendall’s W = 0.290). Post hoc Wilcoxon signed-rank tests with Bonferroni correction showed that only the difference between the Textbook and Clinical Experience reached statistical significance (*p* = 0.032). The differences between ChatGPT and the Textbook (*p* = 0.438) and between ChatGPT and Clinical Experience (*p* = 0.172) were not statistically significant.

Subgroup analysis by training level revealed notable differences between junior and advanced trainees. Junior residents (years 1–2) ranked the LLM-generated content as the most useful source in two-thirds of cases (66.6%), whereas advanced residents (years 3–5) more frequently favored the casebook (43.3%) and their personal experience. This pattern indicates that less experienced trainees may benefit more from structured LLM-generated differential diagnoses, whereas advanced trainees rely more on their accumulated clinical knowledge. Mann–Whitney U test confirmed a significant difference in LLM-dataset ranking between junior and advanced trainees (U = 32, *p* = 0.037), with no significant differences for the other sources (both *p* > 0.05).

Analysis by radiology subspecialty revealed limited variation in ranking patterns across domains. The reference casebook consistently achieved the best mean rank in all subspecialties (range 1.5–1.8), followed by the LLM-generated teaching (range 1.7–2.0), whereas personal clinical experience had the worst mean rank (range 2.3–2.6). The proportion of first-rank assignments for the LLM ranged from 28.0% in Brain and Spine to 42.3% in Chest and Cardiac imaging. In comparison, the casebook was ranked first in 30.8% (Vascular and Interventional) to 53.3% (Pediatric) of cases. Across subspecialties, personal experience was most frequently assigned third rank (59.6–73.0%). When Friedman tests were applied to participant-level aggregated data within each subspecialty, significant differences were observed in Brain and Spine (*p* = 0.009), Chest and Cardiac (*p* = 0.046), and Pediatric (*p* = 0.026) imaging. In all three cases, post hoc analysis confirmed that only the difference between the Textbook and Clinical Experience reached statistical significance after Bonferroni correction (Brain and Spine p.adj = 0.048; Pediatric p.adj = 0.015), while the difference between ChatGPT and Textbook remained non-significant across all subspecialties. No significant differences were found in the remaining six subspecialties.

### 3.3. LLM-Dataset Evaluation

**Figure 6:**
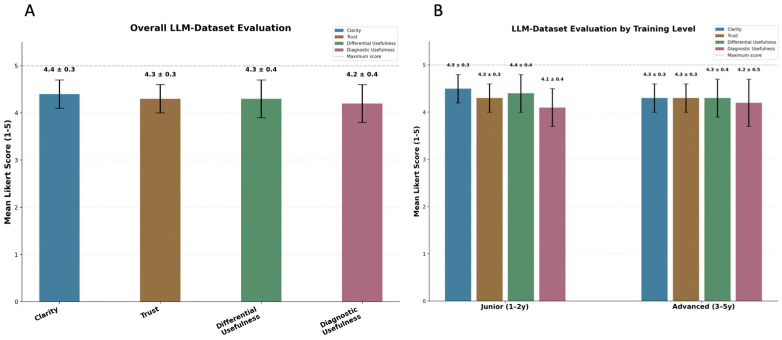
LLM-Dataset Qualitative Evaluation Across Four Performance Domains. (**A**) Overall mean Likert scores (±SD) for Clarity, Trust, Differential Usefulness, and Diagnostic Usefulness across all 2425 evaluations. Scores range from 1 (poor) to 5 (excellent). (**B**) Mean Likert scores (±SD) stratified by training level (junior [1–2 years], advanced [3–5 years]). Error bars represent standard deviation.

**Figure 7:**
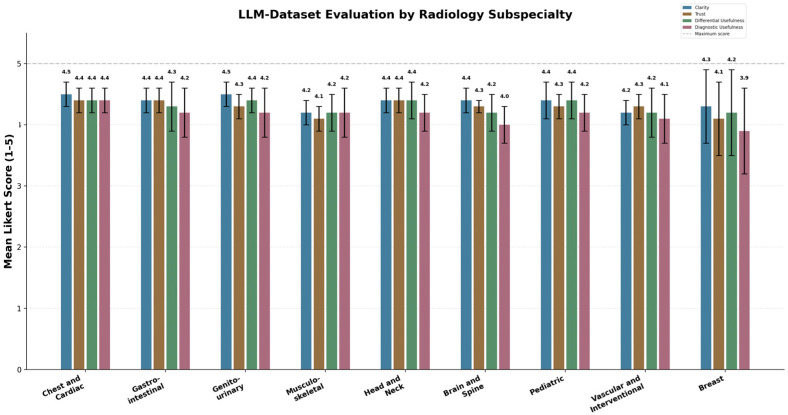
LLM-Dataset Evaluation Across Radiology Subspecialties. Mean Likert scores (±SD) for Clarity, Trust, Differential Usefulness, and Diagnostic Usefulness stratified by radiology subspecialty. Scores range from 1 (poor) to 5 (excellent). Error bars represent standard deviation.

All results for the qualitative evaluation of the LLM-generated dataset are summarized in Table 3, Figure 6 and Figure 7. Overall, the LLM-generated teaching received high mean scores across all four evaluated domains. The highest-rated domain was Clarity, with a mean score of 4.4 ± 0.3, indicating that the text was perceived as clear and well-structured. Trust and Differential Usefulness both received a mean score of 4.3 ± 0.3, reflecting a generally high level of confidence in the content and its value for understanding differential diagnoses. Diagnostic Usefulness, while still positive, received the lowest mean score of the four domains at 4.2 ± 0.4, showing that the LLM output was considered slightly less helpful for confirming the final specific diagnosis. A Friedman test applied to participant-level aggregated data did not confirm significant differences between the four domains (χ^2^ = 5.33, df = 3, *p* = 0.149, Kendall’s W = 0.137). The descriptive hierarchy across domains, with Clarity receiving the highest ratings and Diagnostic Usefulness the lowest, was consistent across participants, but did not reach statistical significance at the participant level.

When comparing the two training levels, both junior and advanced trainees rated the LLM-dataset favorably across all domains, with mean scores consistently above 4.0. Junior trainees assigned the highest mean score to Clarity (4.5 ± 0.3) and the lowest to Diagnostic Usefulness (4.1 ± 0.4). Advanced trainees showed a similar pattern, rating Clarity highest (4.3 ± 0.3) and Diagnostic Usefulness lowest (4.2 ± 0.5). The ratings between the two groups were largely similar, with no substantial deviations in any of the four domains. Mann–Whitney U tests revealed no significant differences between junior and advanced trainees for Clarity (*p* = 0.76), Trust (*p* = 0.70), Differential Usefulness (*p* = 0.94), or Diagnostic Usefulness (*p* = 0.70).

Analysis by radiology subspecialty revealed some variation in the perceived quality of the LLM-generated content. Chest and Cardiac imaging consistently received high ratings across all domains, achieving the highest scores for Diagnostic Usefulness (4.4 ± 0.2) and tying for the highest in other categories. In contrast, Breast imaging received the lowest scores in three out of four domains, including the lowest mean scores for Trust (4.1 ± 0.6) and Diagnostic Usefulness (3.9 ± 0.7). Musculoskeletal imaging and Vascular and Interventional Radiology also showed slightly lower mean scores in several domains compared to other subspecialties. Brain and Spine imaging, while rated well for Clarity (4.4 ± 0.2), received the second-lowest score for Diagnostic Usefulness (4.0 ± 0.3). When Friedman tests were applied to participant-level aggregated data within each subspecialty, significant differences between domains were observed only in Genitourinary imaging (*p* = 0.004), with Clarity rated higher than Diagnostic Usefulness after post hoc correction (p.adj = 0.04). No significant differences were found in the remaining eight subspecialties, likely reflecting the small number of participants per subgroup and the overall uniformly high ratings across domains.

To further illustrate the qualitative characteristics of the LLM-generated content, Supplemental Material File S1 presents one representative best-case and one worst-case example for each subspecialty, together with their mean scores in the four evaluated domains (Clarity, Trust, Differential Usefulness, Diagnostic Usefulness).

## 4. Discussion

We evaluated the educational value of ChatGPT-4o-generated differential diagnoses compared to a standard radiology casebook across nine subspecialties and two training levels. Our findings demonstrate that while traditional casebooks remain the primary educational resource, LLM-generated teaching approaches are comparable in perceived usefulness, indicating significant potential as a supplementary tool for radiology education.

The ranking analysis confirmed statistically significant differences in perceived usefulness across the three sources (*p* = 0.023). The reference casebook achieved the highest mean rank overall, followed closely by the LLM-generated content. This hierarchy suggests that structured educational materials, whether traditional or AI-generated, remain essential for effective learning, as accumulated clinical exposure alone is insufficient, especially during early career stages and particularly in the context of differential diagnosis. The narrow gap between the casebook and LLM rankings (mean ranks of 1.7 vs. 1.8) indicates that LLM-generated content has reached a level of educational quality that trainees perceive as nearly equivalent to established textbook resources for this specific learning objective. Importantly, post hoc analysis revealed that the difference between the Textbook and Clinical Experience was the only statistically significant pairwise comparison (*p* = 0.032), while the difference between LLM and Textbook did not reach significance (*p* = 0.438), further supporting this interpretation. An interesting finding emerged from the subgroup analysis by training level. Junior residents ranked the LLM-generated content as most useful in two-thirds of cases, whereas advanced residents more frequently favored the traditional casebook and their own clinical experience. This divergence may reflect several underlying factors. First, this divergence likely stems from differing baseline knowledge levels and the distinct cognitive demands placed on trainees at different stages of training. Advanced trainees possess a more mature clinical framework, enabling them to rely on their accumulated experience and more readily detect subtle omissions or oversimplifications in the AI’s output. For junior residents, however, the LLM’s highly structured, clear, and bulleted presentation style may significantly reduce cognitive load. Early-stage learners often benefit from a simplified synthesis when first organizing complex differential diagnoses, making the LLM a highly accessible learning tool, whereas advanced residents may perceive the same text-dense format as lacking the depth required for higher-level training. Second, generational differences in technology adoption and familiarity with AI tools may contribute to this pattern, with younger trainees demonstrating greater enthusiasm and comfort with LLM-based learning resources. This is illustrated in a survey of medical students and pathology trainees, where approximately 88% of participants were aware of AI applications in medicine and over 75% expressed a positive attitude toward integrating AI into medical education, with significantly higher enthusiasm observed among students compared to more advanced trainees [28]. Third, this could be due to a selection bias. Only 4 junior participants were recruited, and they may have been inherently more enthusiastic about LLM technology than their 9 advanced peers. Alternatively, the higher LLM rankings among junior trainees may simply reflect a limited ability to detect content deficiencies, as less experienced residents may lack the clinical framework necessary to identify subtle inaccuracies or omissions in AI-generated outputs.

Despite the overall preference for the traditional casebook in the ranking analysis, the LLM-generated dataset received notably high absolute qualitative scores across all four evaluated domains, with mean ratings consistently exceeding 4.0 on a five-point scale. This indicates that radiology trainees appreciate and value this form of educational support, even when they do not rank it as their first choice. While no statistically significant difference was found between the four Likert domains at the participant level (*p* = 0.149), a consistent descriptive hierarchy was observed, with Clarity receiving the highest ratings (mean score 4.4). This result was anticipated, as generating clear, well-structured, and comprehensible text is a well-documented strength of LLMs, particularly when prompted to adopt an educational tone. For example, in a multi-institutional study evaluating the LLM integration into medical curricula, nearly 65% of students perceived these tools as improving clarity and efficiency of studying [29]. Trust received a mean score of 4.3; however, high trust scores reflect persuasive coherence rather than guaranteed factual accuracy. Because highly convincing but incorrect content (hallucinations) poses a significant risk to medical learners, our study partly mitigated this by having participants read the gold-standard casebook immediately prior to evaluating the LLM [30]. However, several important caveats must be acknowledged. First, participants in this study reviewed the reference casebook immediately before evaluating the LLM-generated content for each case. This design feature likely enhanced their ability to detect overt inconsistencies or contradictions between the two sources. Second, participants were radiology trainees rather than experts, and therefore may have lacked the specialized knowledge necessary to identify more subtle inaccuracies or oversimplifications, particularly regarding topics not explicitly covered in the casebook. Third, if the LLM generated plausible-sounding information about aspects of a diagnosis not discussed in the casebook, participants may have accepted this content as it was, leading to an overestimation of trustworthiness. Indeed, in a multi-center survey of 137 general practice trainees evaluating ChatGPT-4o-generated responses, the mean accuracy for detecting AI-generated hallucinations was only 55.0% with a sensitivity of 0.39, indicating that trainees frequently failed to identify incorrect content [31]. These findings underscore the importance of caution when using LLMs as educational resources and reinforce that they should not serve as the sole source of medical knowledge.

To mitigate these hallucination risks and ensure strict factual grounding, future educational tools should leverage Retrieval-Augmented Generation (RAG). As reviewed in healthcare applications [32], RAG frameworks drastically reduce errors by anchoring the LLM to an authoritative database. In a radiology training setting, a RAG system could dynamically retrieve information from vetted textbooks, society guidelines, or institutional teaching files before generating text, effectively blending interactive AI formatting with verified medical accuracy. Furthermore, the possibility of data contamination (where the reference casebook or similar radiology textbooks were included in ChatGPT-4o’s pre-training data) must be considered. While this means the model’s outputs may partly reflect memorized or paraphrased knowledge rather than genuine clinical reasoning, this phenomenon is inherent to the functioning of current LLMs. In the context of medical education and trainee perception benchmarking, the exact computational origin of the knowledge (memorization vs. reasoning) does not diminish the pedagogical utility of the text, provided the final output is accurate, clear, and educationally sound.

A key aspect of radiology education is the distinction between initial image interpretation and the subsequent formulation of a structured differential diagnosis. While image interpretation is inherently visual, the generation and prioritization of differential diagnoses is largely a cognitive and semantic exercise based on structured knowledge. In this context, text-based educational resources, including both traditional casebooks and LLM outputs, primarily address this second component rather than visual pattern recognition. Our study therefore evaluates this downstream cognitive process rather than image interpretation itself, which should be considered when interpreting the generalizability of the findings to radiology training as a whole. Nonetheless, it should be noted that for radiology education in a broader sense, the inability of current LLMs to generate radiological images remains a fundamental limitation, reinforcing the inherently image-centric nature of the specialty.

Both Differential Usefulness and Diagnostic Usefulness received high ratings (4.3 and 4.2, respectively), with no substantial difference between training levels. However, a recurring qualitative comment from participants was that the LLM-generated content occasionally omitted certain differential diagnoses that were included in the casebook. This observation highlights an inherent challenge in differential diagnosis generation: the list of possible diagnoses can be more or less extensive depending on how broadly one defines the clinical scope. The fact that the casebook’s differential diagnoses were curated by expert radiologists provides an intrinsic value and relevance that the LLM has not yet fully matched.

It is highly probable that future generations of radiology trainees will increasingly incorporate LLMs into their learning workflows, regardless of institutional policies or faculty recommendations. As these tools become more sophisticated and accessible, trainees will likely develop expertise in crafting high-quality educational prompts to extract maximum value from these systems. However, two critical limitations must be acknowledged. First, current LLMs cannot reliably generate high-quality, pedagogically appropriate radiological images. This represents a fundamental constraint for radiology education, which is inherently image-centric. Thus, LLM-based learning currently remains a text-limited medium. Second, the risk of hallucinations and inaccuracies, though potentially limited in frequency, remains a serious concern that necessitates cautious integration of these tools. LLMs should be positioned as supplementary resources that complement, rather than replace, established educational material.

The use of a single standardized prompt and a single model configuration (ChatGPT-4o) in our study represents an additional limitation, as this may not reflect the optimal setup for educational content generation. Recent comparative work has demonstrated that LLM performance in radiology tasks varies substantially across models, with significant differences in accuracy and hallucination rates depending on model architecture and configuration [33]. Future studies should systematically evaluate the effect of prompt design and model selection on the educational quality of LLM-generated differential diagnoses.

In alignment with the emerging TRIPOD-LLM (Transparent Reporting of a multivariable model for Individual Prognosis or Diagnosis–Large Language Models) statement established to improve transparency, reproducibility, and methodological clarity in biomedical AI investigations [34], this study satisfies the criteria for an “LLM Evaluation” research design (check-list available as Supplemental Material File S2).

Several limitations of this study warrant acknowledgment. First, the evaluators were radiology trainees rather than subspecialty experts. While this choice reflects the target user population for educational tools, it also means that participants may have lacked the expertise necessary to detect subtle errors or clinically significant hallucinations in the LLM-generated content. Consequently, a systematic, expert-led assessment of factual inaccuracies and omitted diagnoses was beyond the scope of this perception-based study. Second, because participants evaluated cases in a self-directed manner with non-overlapping selections across subspecialties, a formal statistical assessment of inter-rater reliability (such as a fully crossed intraclass correlation coefficient) was not feasible. Furthermore, no repeated measurements were performed, precluding assessment of intra-rater reliability. Third, the evaluation metrics remained qualitative, and no quantitative performance measures, such as correlation with examination scores, were included. Fourth, regarding the small number of independent evaluators, while the total number of evaluations provides sufficient data to assess trainee perceptions across cases and subspecialties, the limited sample size and the small number of junior trainees restrict the statistical robustness of between-group comparisons, particularly the junior versus advanced subgroup analysis, which should be interpreted as exploratory. Finally, selection bias cannot be excluded: participants recruited informally through collegial networks may have had pre-existing enthusiasm for AI technologies, potentially overestimating the perceived educational value of LLM-generated content, and the sample is unlikely to be representative of the broader radiology trainee population. Future work should move beyond subjective ratings and assess whether LLM-based teaching improves measurable outcomes such as examination scores, diagnostic reasoning, reporting quality, or knowledge retention. Such evidence would directly inform the integration of LLMs into radiology training. Ideally, this should be evaluated through prospective, randomized controlled trials comparing three distinct cohorts: casebook-only learning, standalone LLM learning, and a combined casebook-plus-LLM approach, utilizing longitudinal pre- and post-test assessments to quantify true educational impact.

## 5. Conclusions

LLM-generated differential diagnoses are perceived as clear, trustworthy, and useful for educational support by radiology trainees, approaching the usefulness of a standard casebook, particularly among junior trainees. While traditional textbooks remained the primary resource, LLM-generated teaching shows significant promise as a supplementary educational tool. However, known limitations of current LLMs, including the risk of factual inaccuracies and their inability to reproduce image-based teaching that is fundamental to radiology, should be carefully considered. Accordingly, LLMs should complement rather than replace established educational resources.

## Figures and Tables

**Table 1:** Characteristics of Participating Radiologists and Distribution of Evaluated Cases.

Characteristic	Junior Trainees (Year 1–2)	Advanced Trainees (Year 3–5)	All Trainees
Number of Participants (*n*)	4	9	13
Total Questions Evaluated (*n*)	900 (100% of 225)	1525 (75% on average)	2425
Questions Evaluated per Radiologist			
Mean ± SD	225 ± 0	169.4 ± 68.2	186.5 ± 61.8
Range (Min–Max)	225–225	50–225	50–225
Evaluated Questions by Subspecialty, *n* (%)			
Neuroradiology	100 (100%)	200 (88.9%)	300 (92.3%)
Cardiothoracic Imaging	100 (100%)	175 (77.8%)	275 (84.6%)
Genitourinary Imaging	100 (100%)	175 (77.8%)	275 (84.6%)
Musculoskeletal Imaging (MSK)	100 (100%)	175 (77.8%)	275 (84.6%)
Pediatric Imaging	100 (100%)	175 (77.8%)	275 (84.6%)
Gastrointestinal Imaging	100 (100%)	150 (66.7%)	250 (76.9%)
Head and Neck Imaging	100 (100%)	150 (66.7%)	250 (76.9%)
Vascular & Interventional Radiology	100 (100%)	125 (55.6%)	225 (69.2%)
Breast Imaging	100 (100%)	100 (44.4%)	200 (61.5%)

**Table 2:** Perceived usefulness of personal clinical experience, the reference casebook, and ChatGPT-4o-generated teaching for radiology cases. Values show mean ranks ± SD and number (%) of cases assigned to each rank, reported by trainee level and subspecialty. Lower mean ranks indicate higher perceived educational value (1 = most useful, 3 = least useful).

Participant Group	Experience Mean Rank ± SD	Casebook Mean Rank ± SD	LLM Mean Rank ± SD	Experience Rank *n* (%)	Textbook Rank *n* (%)	LLM Rank *n* (%)
All Participants	2.4 ± 0.2	1.7 ± 0.2	1.8 ± 0.3	1st: 607 (24.5%) 2nd: 216 (8.7%) 3rd: 1651 (66.7%)	1st: 987 (39.9%) 2nd: 1223 (49.4%) 3rd: 264 (10.7%)	1st: 892 (36.1%) 2nd: 1036 (41.9%) 3rd: 546 (22.1%)
Per-Training Level Analysis						
Junior (1–2 years)	3.0 ± 0.1	1.7 ± 0.3	1.3 ± 0.3	1st: 6 (0.8%) 2nd: 13 (1.7%) 3rd: 756 (97.5%)	1st: 252 (32.5%) 2nd: 513 (66.2%) 3rd: 10 (1.3%)	1st: 516 (66.6%) 2nd: 250 (32.3%) 3rd: 9 (1.2%)
Advanced (3–5 years)	2.2 ± 0.3	1.7 ± 0.3	2.1 ± 0.3	1st: 601 (35.4%) 2nd: 203 (11.9%) 3rd: 895 (52.7%)	1st: 735 (43.3%) 2nd: 710 (41.8%) 3rd: 254 (14.9%)	1st: 376 (22.1%) 2nd: 786 (46.3%) 3rd: 537 (31.6%)
Per-Subspecialty Analysis						
Chest and Cardiac	2.5 ± 0.2	1.8 ± 0.2	1.7 ± 0.2	1st: 71 (23.7%) 2nd: 22 (7.3%) 3rd: 207 (69.0%)	1st: 102 (34.0%) 2nd: 165 (55.0%) 3rd: 33 (11.0%)	1st: 127 (42.3%) 2nd: 113 (37.7%) 3rd: 60 (20.0%)
Gastrointestinal	2.4 ± 0.2	1.8 ± 0.3	1.8 ± 0.3	1st: 74 (26.9%) 2nd: 23 (8.4%) 3rd: 178 (64.7%)	1st: 95 (34.5%) 2nd: 139 (50.5%) 3rd: 41 (14.9%)	1st: 106 (38.5%) 2nd: 113 (41.1%) 3rd: 56 (20.4%)
Genitourinary	2.5 ± 0.2	1.8 ± 0.3	1.8 ± 0.2	1st: 72 (24.0%) 2nd: 20 (6.7%) 3rd: 208 (69.3%)	1st: 114 (38.0%) 2nd: 146 (48.7%) 3rd: 40 (13.3%)	1st: 114 (38.0%) 2nd: 134 (44.7%) 3rd: 52 (17.3%)
Musculoskeletal	2.4 ± 0.3	1.8 ± 0.2	1.9 ± 0.3	1st: 71 (25.8%) 2nd: 23 (8.4%) 3rd: 181 (65.8%)	1st: 98 (35.6%) 2nd: 147 (53.5%) 3rd: 30 (10.9%)	1st: 105 (38.2%) 2nd: 106 (38.5%) 3rd: 64 (23.3%)
Head and Neck	2.5 ± 0.2	1.7 ± 0.2	1.8 ± 0.2	1st: 73 (26.5%) 2nd: 17 (6.2%) 3rd: 185 (67.3%)	1st: 104 (37.8%) 2nd: 132 (48.0%) 3rd: 39 (14.2%)	1st: 98 (35.6%) 2nd: 126 (45.8%) 3rd: 51 (18.5%)
Brain and Spine	2.4 ± 0.2	1.6 ± 0.2	2.0 ± 0.2	1st: 72 (24.0%) 2nd: 23 (7.7%) 3rd: 205 (68.3%)	1st: 144 (48.0%) 2nd: 134 (44.7%) 3rd: 22 (7.3%)	1st: 84 (28.0%) 2nd: 143 (47.7%) 3rd: 73 (24.3%)
Pediatric	2.6 ± 0.2	1.5 ± 0.2	1.8 ± 0.2	1st: 48 (17.5%) 2nd: 26 (9.5%) 3rd: 200 (73.0%)	1st: 146 (53.3%) 2nd: 112 (40.9%) 3rd: 16 (5.8%)	1st: 93 (33.9%) 2nd: 137 (50.0%) 3rd: 44 (16.1%)
Vascular and Interventional	2.3 ± 0.3	1.8 ± 0.3	1.9 ± 0.3	1st: 82 (32.8%) 2nd: 19 (7.6%) 3rd: 149 (59.6%)	1st: 77 (30.8%) 2nd: 146 (58.4%) 3rd: 27 (10.8%)	1st: 91 (36.4%) 2nd: 84 (33.6%) 3rd: 75 (30.0%)
Breast	2.4 ± 0.2	1.6 ± 0.2	2.0 ± 0.3	1st: 44 (19.6%) 2nd: 43 (19.1%) 3rd: 138 (61.3%)	1st: 107 (47.6%) 2nd: 102 (45.3%) 3rd: 16 (7.1%)	1st: 74 (32.9%) 2nd: 80 (35.6%) 3rd: 71 (31.6%)

**Table 3:** Qualitative evaluation of the LLM-Dataset teaching across four domains: Clarity, Trust, Differential Usefulness, and Diagnostic Usefulness. Values show mean scores ± standard deviation, reported by training level and radiology subspecialty. Ratings are based on a five-point Likert scale where 1 = very poor, 2 = poor, 3 = average, 4 = good, and 5 = very good. Higher scores thus indicate better perceived educational quality.

Participant Group/Subspecialty	Clarity Mean ± SD	Trust Mean ± SD	Differential Usefulness Mean ± SD	Diagnostic Usefulness Mean ± SD
All Participants	4.4 ± 0.3	4.3 ± 0.3	4.3 ± 0.4	4.2 ± 0.4
Per-Training Level Analysis				
Junior (1–2 years)	4.5 ± 0.3	4.3 ± 0.3	4.4 ± 0.4	4.1 ± 0.4
Advanced (3–5 years)	4.3 ± 0.3	4.3 ± 0.3	4.3 ± 0.4	4.2 ± 0.5
Per-Subspecialty Analysis				
Chest and Cardiac	4.5 ± 0.2	4.4 ± 0.2	4.4 ± 0.2	4.4 ± 0.2
Gastrointestinal	4.4 ± 0.2	4.4 ± 0.2	4.3 ± 0.4	4.2 ± 0.4
Genitourinary	4.5 ± 0.2	4.3 ± 0.2	4.4 ± 0.2	4.2 ± 0.4
Musculoskeletal	4.2 ± 0.2	4.1 ± 0.2	4.2 ± 0.3	4.2 ± 0.4
Head and Neck	4.4 ± 0.2	4.4 ± 0.2	4.4 ± 0.3	4.2 ± 0.3
Brain and Spine	4.4 ± 0.2	4.3 ± 0.1	4.2 ± 0.3	4.0 ± 0.3
Pediatric	4.4 ± 0.3	4.3 ± 0.2	4.4 ± 0.3	4.2 ± 0.3
Vascular and Interventional	4.2 ± 0.2	4.3 ± 0.2	4.2 ± 0.4	4.1 ± 0.4
Breast	4.3 ± 0.6	4.1 ± 0.6	4.2 ± 0.7	3.9 ± 0.7

Ratings are based on a five-point Likert scale (1 = very poor, 5 = very good). Values represent the mean score ± standard deviation.

## Data Availability

The original data presented in the study are openly available in Zenodo at https://doi.org/10.5281/zenodo.18667708. The raw evaluation data supporting the conclusions of this article will be made available by the authors on request.

## Supplementary Materials

The following supporting information can be downloaded at: https://www.mdpi.com/article/10.3390/diagnostics16132009/s1, File S1: Best-case and worst-case examples of LLM-generated differential diagnosis teaching for each of the nine radiology subspecialties. File S2: TRIPOD-LLM checklist for the present study.

## Abbreviations

The following abbreviations are used in this manuscript:

AI	Artificial Intelligence
LLM	Large Language Model
ChatGPT	Chat Generative Pre-trained Transformer
GPT-4o	Generative Pre-trained Transformer 4 omni
IRB	Institutional Review Board
IQR	Interquartile Range
SD	Standard deviation
MSK	Musculoskeletal
RAG	Retrieval-Augmented Generation
