# Supplementary material for: Large Language Model-Generated Differential Diagnoses in Radiology Education: Comparison with a Standard Casebook
Source: Diagnostics (Basel). 2026 Jun 27;16(13):2009. doi: 10.3390/diagnostics16132009 (PMC13360087; doi:10.3390/diagnostics16132009)
Supplement: Supplementary file 1 [file diagnostics-16-02009-s001.zip › Supplemental Material File S1-Examples.pdf]

Category: Chest and Cardiac

Best Case – Case 20 (Clarity: 4.6, Trust: 4.5, Differential Usefulness: 4.8, Diagnostic Usefulness: 4.7)

Case 20

Pleural-Based Mass – Radiological Summary

Definition

A pleural-based mass is an abnormal soft-tissue lesion arising from the pleura, which can be **benign or malignant**, primary or secondary. Imaging evaluation focuses on **location, morphology, enhancement, calcification, and associated pleural or lung findings**.

CT Chest Features (Key Imaging Clues)

✓ Location:

1. Arises from **pleura**, often broad-based against the chest wall.
2. May cause **pleural effusion, thickening, or calcification**.

✓ Morphology:

1. **Well-defined, lobulated** (benign or malignant).
2. **Invasive, irregular margins** (malignant).

✓ Enhancement:

1. **Heterogeneous, necrotic, or solid enhancement** → Suggests malignancy.
2. **Homogeneous, non-aggressive** → Benign lesion.

✓ Calcifications:

1. **Diffuse or nodular** → Mesothelioma, old TB, or prior asbestos exposure.
2. **Peripheral eggshell calcifications** → Solitary fibrous tumor, old infections.

✓ Associated Findings:

1. **Pleural effusion** (exudative) → Suggests malignancy (esp. mesothelioma).

2. **Rib destruction/chest wall invasion** → Aggressive tumor (sarcoma, mesothelioma).

Differential Diagnosis of Pleural-Based Masses

1. Malignant Causes

✅ **Malignant Pleural Mesothelioma (MPM) (Most Common Primary Malignancy)**

1. **CT Features:** Diffuse, nodular pleural thickening ± calcifications.
2. **Associated with:** Asbestos exposure, unilateral pleural effusion.
3. **Pearl:** Involves **pleural fissures, chest wall invasion**.

✅ **Metastatic Pleural Disease (Most Common Overall Malignancy)**

1. **CT Features:** Multiple pleural nodules, effusion, lung/breast/ovarian malignancies.
2. **Pearl:** History of **lung, breast, GI, ovarian carcinoma**.

✅ **Solitary Fibrous Tumor (SFT) (Benign but Can Be Malignant)**

1. **CT Features:** Well-defined, lobulated, pleural-based mass, ± calcifications.
2. **Pearl: Does NOT cause pleural effusion**, often incidental.
3. **Hypervascular with strong enhancement**.

✅ **Pleural Lymphoma**

1. **CT Features:** Diffuse pleural thickening, ± mediastinal lymphadenopathy.
2. **Pearl:** History of **chronic inflammation (e.g., TB, chronic effusion)**.

✅ **Synovial Sarcoma (Rare, Aggressive, Soft-Tissue Malignancy)**

1. **CT Features:** Large, heterogeneously enhancing, invading chest wall.
2. **Pearl:** Seen in **younger adults**, may mimic mesothelioma.

---

2. Benign Causes

✅ **Fibrous Pleural Tumor (SFT – Benign Form)**

1. **CT Features:** Well-defined, non-invasive, slow-growing, ± calcifications.

2. **Pearl:** Can cause **hypoglycemia (Doege-Potter syndrome)**.

✅ **Pleural Lipoma**

1. **CT Features:** Homogeneous **fat attenuation (-50 to -100 HU)**.
2. **Pearl:** No enhancement, no pleural effusion.

✅ **Pleural Plaques (Asbestos-Related Benign Disease)**

1. **CT Features:** Thick, **calcified plaques along diaphragm and chest wall**.
2. **Pearl:** No mass effect, no effusion.

Imaging Approach

✔ **CXR:** May show a **pleural-based opacity with obtuse angles**.
✔ **CT Chest (Gold Standard):** Characterizes mass (enhancement, calcification, invasion).
✔ **MRI:** Differentiates **soft-tissue components vs. fibrosis**.
✔ **PET-CT:** Differentiates **benign vs. malignant** (FDG avidity).

Key Exam Pearls

🔹 **Diffuse pleural thickening + history of asbestos** → Mesothelioma.
🔹 **Multiple pleural nodules + primary cancer history** → Metastases.
🔹 **Solitary well-defined pleural mass + no effusion** → Solitary fibrous tumor.
🔹 **Pleural mass + bone invasion** → Synovial sarcoma or aggressive malignancy.
🔹 **Pleural fat density lesion** → Lipoma (benign).

Would you like a **table for rapid comparison of key features**? 🚀

Worst Case – Case 23 (Clarity: 4.3, Trust: 4.5, Differential Usefulness: 3.8, Diagnostic Usefulness: 3.9)

Case 23

Cardiac Mass – Radiological Summary

Definition

A **cardiac mass** refers to any abnormal lesion within or adjacent to the heart, which may be **tumorous (benign or malignant), thrombotic, or infectious**. Imaging assessment involves **echocardiography, cardiac CT, and cardiac MRI** for localization, tissue characterization, and differentiation from normal structures.

Imaging Approach

1. **Echocardiography (TTE/TEE)**
    1. First-line for localization, mobility, and hemodynamic impact.
    2. TEE is superior for **atrial masses, small thrombi, and posterior lesions**.
2. **Cardiac CT**
    1. Evaluates **calcification, vascularity, pericardial invasion**.
    2. Useful for differentiating **thrombus vs tumor**.
3. **Cardiac MRI**
    1. Best for **tissue characterization** (T1, T2, perfusion, LGE).
    2. Helps differentiate **benign vs malignant** masses.

Common Types of Cardiac Masses

1. Benign Primary Tumors (75-80%)

✅ **Myxoma (Most Common Benign Tumor, 50%)**

1. **Location: Left atrium (75%)**, attached to fossa ovalis.
2. **CT/MRI:** Low T1, high T2 signal, heterogeneous post-contrast enhancement.
3. **Pearl: Can cause embolism, mitral obstruction, or constitutional symptoms**.

✅ **Papillary Fibroelastoma (Most Common Valve Tumor)**

1. **Location:** Aortic or mitral valve.
2. **ECHO:** Small, mobile, frond-like mass.
3. **Pearl: Risk of embolism**, typically detected incidentally.

✅ **Lipoma**

1. **CT:** Fat attenuation (-50 to -100 HU).
2. **MRI:** High T1 signal, nulls on fat saturation.
3. **Pearl: Well-circumscribed, often asymptomatic**.

✅ **Rhabdomyoma (Most Common in Children, Tuberous Sclerosis)**

1. **Location:** Ventricles > atria.
2. **MRI:** Isointense to myocardium on T1, hyperintense on T2.
3. **Pearl: Spontaneous regression in infants**.

✅ **Hemangioma & Fibroma**

1. **Fibroma:** T1/T2 **hypointense**, intense LGE.
2. **Hemangioma:** Highly vascular, intense enhancement.

---

2. Malignant Primary Tumors (Rare, but Aggressive)

✅ **Angiosarcoma (Most Common Primary Malignant Tumor)**

1. **Location: Right atrium**, infiltrating myocardium.
2. **MRI:** Heterogeneous mass, T2 hyperintense, avid enhancement.
3. **Pearl: Hemorrhage, necrosis, right heart failure symptoms**.

✅ **Rhabdomyosarcoma (Most Common in Children)**

1. **MRI:** Infiltrative soft tissue mass, variable enhancement.

✅ **Mesothelioma (Pericardial Origin)**

1. **CT/MRI:** Circumferential pericardial thickening ± effusion.

---

3. Cardiac Thrombus (Common Mimic of Tumors)

✅ **Most Common Locations:**

1. **Left atrial appendage** (AF, mitral stenosis).
2. **LV apex** (Post-MI, dilated cardiomyopathy).

✅ **Imaging Clues:**

1. **Non-enhancing mass** (no first-pass or delayed contrast uptake).
2. **ECHO:** Sessile, hypoechoic mass ± spontaneous echo contrast.
3. **MRI:** Dark on T1/T2, no LGE.

---

4. Metastatic Disease (Most Common Cardiac Malignancy)

✅ **Sources:** Lung, breast, melanoma, lymphoma, RCC.
✅ **CT/MRI:** Multiple enhancing lesions ± pericardial effusion.
✅ **Pearl:** Metastases are **40x more common than primary malignancies**.

Key Exam Pearls

- **Left atrial mass attached to septum → Myxoma**.
- **Mobile valve mass in elderly → Papillary fibroelastoma**.
- **Fat-density mass → Lipoma**.
- **Right atrial infiltrative tumor with necrosis → Angiosarcoma**.
- **Non-enhancing mass in LV apex → Thrombus**.
- **Multiple pericardial/epicardial nodules → Metastases**.

Would you like a **table with differentiating imaging features**? 🚀

Category: GastroIntestinal

Best Case – Case 29 (Clarity: 4.7, Trust: 4.9, Differential Usefulness: 4.5, Diagnostic Usefulness: 4.6)

Case 29

Solitary Hypodense, Hypovascular Liver Mass – Exam-Oriented Radiological Summary

1. Key Considerations

1. **Hypodense on CT → Broad differential, must correlate with clinical context.**
2. **Hypovascular (No early arterial enhancement) → Excludes hypervascular lesions (HCC, FNH, adenoma).**
3. **Evaluate lesion morphology, margins, enhancement, and associated findings.**

---

2. Differential Diagnosis Based on Key Features

---

3. Imaging Approach

CT (Best for Initial Assessment)

1. **Metastases:** Multiple lesions, hypoenhancing, may have central necrosis.

2. **Cholangiocarcinoma:** Capsular retraction, progressive delayed enhancement.
3. **Abscess:** Peripheral rim enhancement, gas bubbles (bacterial).
4. **Cyst:** Homogeneous, water attenuation, no enhancement.

MRI (Best for Characterization of Cysts vs. Solid Lesions)

1. **Cysts:** T2 hyperintense, no enhancement.
2. **Cholangiocarcinoma:** T2 hyperintense, delayed enhancement, fibrosis.
3. **Abscess:** T2 hyperintense, diffusion restriction, rim enhancement.
4. **Lymphoma:** Hypointense on T1, hyperintense on T2, restricted diffusion.

PET-CT (For Malignant Suspicion, Lymphoma, or Metastases)

1. **Hypermetabolic = Malignancy (Metastases, Cholangiocarcinoma, Lymphoma).**
2. **Cold lesion = Cyst, hemangioma.**

---

4. Key Exam Points

1. **Most common hypodense liver lesion → Metastasis (look for primary).**
2. **Water-density, non-enhancing = Cyst.**
3. **Peripheral enhancement + gas = Abscess.**
4. **Delayed enhancement + capsular retraction = Cholangiocarcinoma.**
5. **Solitary hypoattenuating lesion in immunocompromised → Consider fungal abscess or lymphoma.**

Would you like a quick decision tree for approaching hypodense liver lesions?

Worst Case – Case 41 (Clarity: 3.9, Trust: 4.0, Differential Usefulness: 3.3, Diagnostic Usefulness: 3.3)

Case 41

Radiological Summary: Mesenteric Mass

Differential Diagnosis (Based on Imaging Features)

1. **Benign Lesions**
    1. **Mesenteric cyst**: Well-defined, thin-walled, fluid attenuation on CT, no enhancement, may have septations.

    2. **Sclerosing mesenteritis**: Fatty mesenteric mass with surrounding soft-tissue nodularity and a "fat ring sign" (preserved fat around vessels).
    3. **Desmoid tumor**: Well-defined, soft-tissue mass without calcification, no necrosis, may cause mesenteric retraction.
2. **Malignant Lesions**

    1. **Lymphoma**: Large, soft-tissue mass, homogeneous, encases vessels without occlusion ("sandwich sign"), no calcification.
    2. **Carcinoid tumor metastasis**: Desmoplastic reaction with calcifications, mesenteric retraction, tethering of bowel loops, hypervascular primary (often ileal).
    3. **Gastrointestinal stromal tumor (GIST)**: Large, necrotic mass, heterogeneous enhancement, often exophytic from bowel.
    4. **Peritoneal carcinomatosis**: Multiple soft-tissue implants, omental caking, ascites (often from ovarian, gastric, or colon cancer).

Key Imaging Modalities

1. **CT with contrast (Primary modality)**: Evaluates size, enhancement pattern, calcifications, vascular involvement.
2. **MRI**: Useful for soft-tissue characterization in indeterminate cases.
3. **PET-CT**: For metabolic activity assessment (lymphoma, metastases).

Exam Tip

1. **Encasement without obstruction → Think lymphoma.**
2. **Calcifications with desmoplastic reaction → Carcinoid metastasis.**
3. **Fatty mass with a "fat ring sign" → Sclerosing mesenteritis.**

Would you like a quick case-based approach for exam scenarios?

Category: Genitourinary

Best Case – Case 67 (Clarity: 4.7, Trust: 4.7, Differential Usefulness: 4.8, Diagnostic Usefulness: 4.6)

Case 67

Perinephric Fluid Collection – Exam-Oriented Radiology Summary

Differential Diagnosis:

1. Hemorrhagic Collections

4. **Renal Trauma (Perinephric Hematoma)**
    1. **CT:** Hyperdense (acute), evolving to mixed/lower density
    2. **No contrast extravasation unless active bleeding**
5. **Spontaneous Renal Hemorrhage (Wunderlich Syndrome)**
    1. Causes: Renal tumors (e.g., RCC, AML), coagulopathy, vasculitis
    2. **CT:** Perinephric hematoma ± active contrast extravasation
6. **Anticoagulation-Related Hematoma**
    1. Similar to spontaneous hemorrhage, history of anticoagulant use

2. Infectious / Inflammatory Collections

7. **Perinephric Abscess**
    1. Complication of **pyelonephritis, renal abscess, or post-surgical infection**
    2. **CT:** Perinephric fluid collection with **rim enhancement**, ± gas formation (if emphysematous)
    3. **US:** Complex cystic mass, internal debris, ± gas
8. **Pyonephrosis (Infected Hydronephrosis)**
    1. **CT:** Dilated collecting system with debris/fluid level, thickened walls
    2. **US:** Echogenic debris within hydronephrotic kidney

3. Urinary Collections (Urine Leaks / Urinoma)

9. **Post-traumatic or post-surgical urinoma** (e.g., after renal biopsy, partial nephrectomy)
    1. **CT (Excretory Phase):** Fluid collection with **delayed contrast extravasation**
    2. **US:** Anechoic perinephric collection

4. Lymphatic & Miscellaneous Collections

10. **Lymphocele** (Post-renal transplant or surgery)
    1. **CT:** Well-defined, **non-enhancing**, simple fluid collection
    2. **US:** Anechoic, compressible, no internal debris
11. **Perinephric Pseudocyst (Chronic)**
    1. Chronic perinephric fluid collection from prior hemorrhage/infection
    2. Fibrous encapsulation, no contrast enhancement

Key Imaging Features & Modalities:

1. CT (Preferred Modality for Perinephric Collections)

12. **Hemorrhage:** Hyperdense (acute), no enhancement
13. **Abscess:** Rim-enhancing collection ± gas
14. **Urinoma:** Low-density collection, contrast extravasation in delayed phase
15. **Lymphocele:** Simple fluid density, no enhancement

2. Ultrasound (For Initial Assessment & Follow-Up)

16. **Hematoma:** Hyperechoic (acute), becomes anechoic/hypoechoic over time
17. **Abscess:** Thick-walled, internal debris, possible gas artifacts
18. **Urinoma / Lymphocele:** Simple anechoic fluid collection

3. MRI (Adjunct for Problem-Solving)

1. Useful for complex collections when CT findings are inconclusive
2. **Hematoma:** T1 hyperintense (acute), T2 hyperintense (chronic)
3. **Abscess:** Rim-enhancing lesion with restricted diffusion

---

Key Differentiating Points:

Exam Pearls:

✅ **Trauma → Think hematoma (acute hyperdensity on CT)**

✅ **Post-surgical perinephric fluid → Urinoma (delayed contrast extravasation)**

✅ **Pyelonephritis complication → Perinephric abscess (rim enhancement, possible gas)**

✅ **Renal transplant → Lymphocele (simple fluid, no enhancement)**

Would you like another genitourinary topic summarized?

Worst Case – Case 64 (Clarity: 4.3, Trust: 4.3, Differential Usefulness: 3.9, Diagnostic Usefulness: 3.2)

Case 64

Renal Migration Anomaly – Exam-Oriented Radiology Summary

Definition:

Renal migration anomalies occur due to **failure of normal cranial migration of the kidneys** during embryogenesis, leading to abnormal positioning.

Types & Key Imaging Features:

1. Pelvic Kidney (Most Common)

1. Kidney remains in the **pelvis due to migration failure**
2. **CT / Ultrasound:**
    1. Kidney located in pelvis, often smaller than normal
    2. Short ureter, may cross iliac vessels
    3. Normal renal enhancement
    4. **Complications:** Hydronephrosis, infection, stones

2. Crossed Fused Ectopia

1. **Both kidneys on one side**, with **fusion** of the ectopic kidney to the normally positioned kidney
2. **CT Urography:**
    1. Ectopic kidney fused to lower pole of normal kidney
    2. **Single ureteric insertion per kidney**
    3. **DRAINAGE ISSUES → Hydronephrosis risk**

3. Horseshoe Kidney

1. **Fusion of lower poles (90%)** across midline, at L3-L5 level
2. **CT / MRI:**
    1. **"U"-shaped isthmus crossing midline** anterior to the aorta
    2. **Malrotation** (hilum faces anteriorly)
    3. **Increased stone & infection risk** due to abnormal drainage
    4. **Association:** Wilms tumor, renal cell carcinoma (RCC)

4. Thoracic Kidney (Rare)

1. Kidney migrates **above diaphragm**, herniating through the foramen of Bochdalek
2. **CT / MRI:**
    1. **Renal tissue in thoracic cavity**
    2. Herniated through diaphragm, often asymptomatic

Key Differentiating Points:

Exam Pearls:
✅ **Pelvic kidney = Most common migration anomaly** → Short ureter, risk of obstruction
✅ **Horseshoe kidney → "U"-shaped isthmus, malrotation, stone risk**
✅ **Crossed fused ectopia = Both kidneys on one side, single ureter insertion per kidney**
✅ **Thoracic kidney → Herniation through Bochdalek foramen, rare**

Would you like a summary of another genitourinary anomaly?

Category: Musculoskeletal

Best Case – Case 100 (Clarity: 4.5, Trust: 4.4, Differential Usefulness: 4.5, Diagnostic Usefulness: 4.6)

Case 100

Epiphyseal Equivalent Lucent Lesions – Exam-Oriented Radiology Summary

Definition:

Epiphyseal equivalent lesions occur in **epiphyses, apophyses, and secondary ossification centers** in skeletally immature patients.

Differential Diagnosis (FAMEE Mnemonic):

Key Imaging Features per Modality:

1. **X-ray:** Well-defined lucent lesion in epiphyseal or equivalent location

2. **MRI:**
    1. **T1:** Low to intermediate signal
    2. **T2:** High signal (except fibrous dysplasia, which is intermediate)
    3. **Post-contrast:** Variable enhancement (solid components enhance)
3. **CT:** Best for cortical integrity and matrix characterization

---

Exam Pearls:

✅ **Chondroblastoma = Classic epiphyseal lesion in adolescents**
✅ **GCT = Epiphyseal lesion in adults (after physis closure)**
✅ **ABC = Expansile lesion with fluid-fluid levels**
✅ **Fibrous dysplasia = "Ground-glass" matrix, no periosteal reaction**
✅ **LCH = Lytic lesion with variable sclerosis, may mimic infection**

Would you like another musculoskeletal topic summarized?

Worst Case – Case 94 (Clarity: 4.0, Trust: 4.0, Differential Usefulness: 3.8, Diagnostic Usefulness: 3.5)

Case 94

Radiological Summary: Posterior Element Lytic Lesion (Exam-Oriented Approach)

Definition:

A **posterior element lytic lesion** refers to a **destructive or expansile lesion** involving the **lamina, pedicle, spinous process, transverse process, or facets** of the vertebrae. The differential diagnosis depends on **age, lesion characteristics, and associated findings**.

Key Differential Diagnoses Based on Etiology

Key Imaging Modalities & Findings

1. X-ray (First-Line Investigation)

1. **"Winking owl" sign → Pedicle metastasis.**

2. **Expansile lytic lesion with sclerosis → Osteoblastoma.**
3. **Collapsed vertebra (vertebra plana) → LCH, Myeloma.**

2. CT (Better for Bone Detail)

1. **Fluid-fluid levels → ABC.**
2. **Cortical expansion, matrix mineralization → Osteoblastoma.**
3. **Vertebral destruction with abscess → Tuberculosis.**

3. MRI (Soft Tissue & Marrow Involvement)

1. **Low T1, high T2 with contrast enhancement → Infection, Tumor.**
2. **Soft tissue mass with marrow edema → Ewing Sarcoma, Metastasis.**

Exam-Oriented Diagnostic Approach:

1. **Assess for Aggressive vs. Non-Aggressive Features:**
   1. **Expansile, well-defined → Osteoblastoma, ABC.**
   2. **Aggressive, permeative destruction → Metastasis, Ewing Sarcoma.**
2. **Check for Associated Soft Tissue Mass or Fluid Levels:**
   1. **Soft tissue mass → Malignancy (Ewing, Metastasis).**
   2. **Fluid-fluid levels → ABC.**
3. **Evaluate Age & Systemic Signs:**
   1. **Young child, vertebra plana → LCH.**
   2. **Elderly with multiple lesions → Myeloma, Metastases.**
4. **Confirmatory Workup:**
   1. **Biopsy if malignancy or infection suspected.**
   2. **Serum markers: ALP (osteoblastoma), CRP/ESR (infection), SPEP (myeloma).**

A **posterior element lytic lesion** is a **critical imaging finding** requiring differentiation between **benign, malignant, and infectious causes** through **systematic imaging analysis and clinical correlation**.

Category: Head and Neck

Best Case – Case 114 (Clarity: 4.7, Trust: 4.7, Differential Usefulness: 4.6, Diagnostic Usefulness: 4.5)

Case 114

Radiological Summary: Globe Lesion in a Child

Definition

A **globe lesion in a child** refers to an intraocular or periorbital abnormality involving the **retina, vitreous, choroid, sclera, or optic nerve**. The differential diagnosis includes **neoplastic, congenital, inflammatory, and post-traumatic causes**.

Imaging Modality & Key Findings

1. Ultrasound (US) – First-line for Ocular Pathology

5. **Highly sensitive for calcifications (retinoblastoma)**.
6. **B-scan**: Differentiates solid vs. cystic masses.
7. **A-scan**: Determines internal reflectivity.

2. MRI Orbit (Preferred for Soft Tissue & Extent of Lesion)

8. **T1/T2 signal characteristics**.
9. **Post-contrast enhancement**.
10. **DWI restriction (e.g., retinoblastoma)**.
11. **Optic nerve involvement**.

3. CT Orbit (Best for Calcifications & Bony Involvement)

12. **Detects intraocular or intratumoral calcifications (retinoblastoma)**.
13. **Evaluates globe size (microphthalmia, coloboma)**.

Differential Diagnosis Based on Imaging Features

1. Retinoblastoma (Most Common Intraocular Malignancy in Children)

14. **CT**: Intraocular **calcifications** (95% cases).
15. **MRI**:
    1. **T1 hypointense, T2 hyperintense**, moderate enhancement.
    2. **Restricted diffusion on DWI**.
    3. **Optic nerve, choroidal, or extraocular spread possible**.

2. Persistent Fetal Vasculature (PFV) (Persistent Hyperplastic Primary Vitreous - PHPV)

16. **Microphthalmia with elongated hyperintense stalk (T1/T2 MRI)**.
17. **No calcifications (helps differentiate from retinoblastoma)**.
18. Associated with congenital cataract, retinal detachment.

3. Coats Disease (Exudative Retinal Vasculopathy, Unilateral, M>F)

19. **Retinal detachment + Subretinal exudates**.
20. **No intraocular calcifications**.
21. **T1 hyperintense lipid deposition within subretinal space**.

4. Retinopathy of Prematurity (ROP) (Neonatal with Oxygen Therapy History)

22. **Bilateral retinal detachment**.
23. **No mass or calcifications**.

5. Optic Nerve Glioma (Association with NF1, Slow-Growing Tumor)

1. **Fusiform optic nerve thickening**.
2. **T2 hyperintensity, variable enhancement**.
3. **No calcifications**.

6. Ocular Trauma (Retinal Detachment, Vitreous Hemorrhage)

1. **Retinal detachment**: **V-shaped hyperintense membrane** on MRI.

2. **Vitreous hemorrhage**: **Acute (T1 hyperintense), Chronic (T2 dark due to hemosiderin)**.

Key Imaging Approach for Exam Cases

1. **Is there intraocular calcification?**
    1. **Yes → Retinoblastoma**.
    2. **No → Consider Coats disease, PFV, or trauma**.
2. **Is there retinal detachment?**
    1. **Yes + Exudates → Coats disease**.
    2. **Yes + Microphthalmia → PFV**.
    3. **Yes + Prematurity → ROP**.
3. **Is the optic nerve involved?**
    1. **Yes → Optic nerve glioma**.

Key Exam Pearls

1. **Retinoblastoma = Most common intraocular tumor, intraocular calcifications are key on CT**.
2. **PFV = Microphthalmia + Persistent Retrolental Stalk (No calcifications)**.
3. **Coats Disease = Retinal detachment + Lipid-rich exudates (Unilateral, No calcifications)**.
4. **ROP = Prematurity history + Bilateral retinal detachment**.
5. **Optic Nerve Glioma = Fusiform optic nerve thickening, NF1 association**.

Would you like additional case-based questions for further exam preparation?

Worst Case – Case 113 (Clarity: 4.1, Trust: 4.1, Differential Usefulness: 3.4, Diagnostic Usefulness: 3.2)

Case 113

Radiological Summary: Vascular Injury to the Neck

Definition

Vascular injury to the neck involves trauma to the **carotid or vertebral arteries, jugular veins**, or associated structures due to **penetrating (gunshot, stab) or blunt trauma (hyperextension, direct impact, strangulation)**. This can lead to **dissection, occlusion, pseudoaneurysm, arteriovenous fistula (AVF), or active hemorrhage**.

Imaging Modality & Key Findings

1. CT Angiography (CTA) Neck (First-line Imaging for Vascular Injury)

1. **Lumen irregularity** → Suggests **intimal injury or dissection**.
2. **Abrupt vessel cutoff** → Suggests **occlusion or thrombosis**.
3. **Contrast extravasation** → Indicates **active bleeding**.
4. **Pseudoaneurysm** → Focal contrast-filled outpouching.
5. **AVF** → Early venous filling.
6. **Soft tissue hematoma** → May compress vascular structures.

2. Digital Subtraction Angiography (DSA) – Gold Standard (If Intervention Needed)

1. **Higher sensitivity for subtle injuries**.
2. **Allows for therapeutic intervention (stenting, embolization)**.

3. Doppler Ultrasound (Limited Role in Acute Trauma, Used for Stable Cases)

1. Can detect **occlusion, intimal flap, pseudoaneurysm, AVF**.
2. **Operator-dependent**, limited by overlying soft tissue and gas.

Classification of Vascular Injury (BCVI - Blunt Cerebrovascular Injury Grading, Biffl Scale for Carotid/Vertebral Artery Injury)

Common Mechanisms & Imaging Findings

1. Carotid Artery Dissection (Blunt Trauma, Hyperextension-Rotation Injury)

1. **String sign** (narrowed lumen from intimal tear).
2. **Tapered occlusion**.
3. **Intimal flap** or **double lumen**.
4. **Fat-suppressed T1 MRI**: Crescentic **hyperintensity** (intramural hematoma).

2. Vertebral Artery Dissection (Cervical Spine Fractures, Whiplash Injury)

1. Similar findings to carotid dissection.
2. **High association with C1-C2 fractures** (e.g., Hangman's fracture).

3. Pseudoaneurysm (Penetrating Trauma, Iatrogenic Injury)

1. Well-defined contrast-filled outpouching **without normal vessel wall**.
2. May enlarge and rupture.

4. Arteriovenous Fistula (AVF) (High-Velocity Penetrating Trauma)

3. Early venous filling of jugular vein in arterial phase.
4. Abnormal turbulent flow at fistulous connection.

5. Active Hemorrhage (Penetrating Trauma, Blunt Trauma with Vascular Rupture)

5. Contrast extravasation outside vascular structures.
6. Expanding hematoma or mass effect on adjacent tissues.

Key Imaging Approach for Exam Cases

7. **Is there vessel irregularity or stenosis?**
    1. **Yes → Think dissection or intimal injury**.
8. **Is there a well-defined contrast-filled outpouching?**
    1. **Yes → Pseudoaneurysm**.
9. **Is there early venous filling in arterial phase?**

    1. **Yes → AV fistula**.

10. **Is there contrast extravasation?**

    1. **Yes → Active hemorrhage (emergency)**.

Key Exam Pearls

11. **Blunt trauma + stroke symptoms = Suspect carotid/vertebral dissection** (CTA needed).
12. **Expanding neck hematoma + pulsatile mass = Pseudoaneurysm or AVF**.
13. **C1-C2 fractures → High risk of vertebral artery dissection**.
14. **Active extravasation on CTA = Emergency intervention required**.
15. **If CTA negative but high suspicion remains → Consider DSA (gold standard)**.

Would you like additional case-based questions or quick review flashcards for further preparation?

Category: Pediatric

Best Case – Case 166 (Clarity: 4.7, Trust: 4.6, Differential Usefulness: 4.7, Diagnostic Usefulness: 4.8)

Case 166

Posterior Vertebral Body Scalloping – Pediatric Radiology Summary

Definition

Posterior vertebral body scalloping refers to excessive concavity of the posterior vertebral cortex, best seen on lateral spine radiographs. It may be physiological or pathological.

Differential Diagnosis

1. **Physiological Scalloping**
    1. Mild, smooth concavity at multiple levels.
    2. Seen in normal children due to dural expansion.
2. **Pathological Causes**

A. Increased Intraspinal Pressure (*Dural ectasia, CSF pulsations*)

1. **Neurofibromatosis Type 1 (NF1)** (Most common) → Associated with ribbon ribs, lateral thoracic meningoceles.
2. **Achondroplasia** → Small foramen magnum, narrowing of interpedicular distance.
3. **Marfan Syndrome / Connective Tissue Disorders** → Associated with dural ectasia, aortic aneurysms.

B. Intraspinal Mass Lesions (*Chronic pressure erosion*)

4. **Spinal tumors (Ependymoma, Astrocytoma, Meningocele, Lipoma)** → Smooth or focal scalloping.

C. Congenital / Dysplastic Disorders

5. **Mucopolysaccharidoses (MPS, e.g., Hurler, Morquio syndromes)** → Ovoid vertebrae, anterior beaking, thickened ribs.
6. **Congenital spinal dysraphism (e.g., tethered cord, lipomyelomeningocele)** → Scalloping with associated spinal abnormalities.

Imaging Findings

1. Lateral Spine X-ray:

1. **Smooth, diffuse scalloping → Dural ectasia (NF1, Marfan, Achondroplasia).**
2. **Focal, irregular scalloping → Intraspinal tumor or mass.**
3. **Scalloping with anterior vertebral beaking → MPS.**

2. MRI Spine (Best for Intraspinal Pathology):

1. **Dural ectasia → Posterior vertebral body thinning, CSF-filled outpouching.**
2. **Intraspinal tumor → Mass effect with cord displacement.**
3. **Neurofibromas → Target sign, T2 hyperintense masses.**

Key Exam Pearls

1. **Smooth scalloping, multiple levels → Think NF1, Marfan, or dural ectasia.**
2. **Focal scalloping → Consider intraspinal mass (tumor, meningocele).**
3. **Anterior vertebral beaking + posterior scalloping → MPS.**
4. **Associated with achondroplasia → Small foramen magnum, spinal stenosis.**

Would you like further differentiation of dural ectasia vs. mass-related causes?

Worst Case – Case 175 (Clarity: 4.1, Trust: 3.9, Differential Usefulness: 3.8, Diagnostic Usefulness: 3.4)

Case 175

Esophageal Obstruction in a Neonate – Exam-Oriented Radiological Summary

Key Differential Diagnosis (Mnemonic: "A-TEAM")

5. **A**tresia (Esophageal Atresia ± Tracheoesophageal Fistula) → Most common
6. **T**racheoesophageal Fistula (Without Atresia – H-type)
7. **E**sophageal Webs/Rings (Rare, associated with syndromes)
8. **A**berrant Right Subclavian Artery (Dysphagia Lusoria)
9. **M**asses (Intrinsic: Duplication cyst, Extrinsic: Vascular ring/slings, teratoma)

---

1. Esophageal Atresia ± Tracheoesophageal Fistula (EA ± TEF)

✅ **Types:**

10. **Type C (90%) → Proximal atresia + distal TEF**
11. **Type A (8%)** → Pure atresia (no fistula)
12. **Type E (H-type, 4%)** → No atresia, isolated TEF

✅ **Imaging Findings:**

13. **CXR with NG Tube**:
    1. Coiled **NG tube in upper pouch** (proximal atresia)
    2. No gas in abdomen (pure atresia, Type A)
    3. **Gas in abdomen** (TEF present, Type C/E)
14. **Fluoroscopy (Esophagogram)**:
    1. Contrast stops at atresia (avoid if suspected TEF to prevent aspiration)

✅ **Management:**

15. **Surgical correction** (emergent for significant TEF)

---

2. Tracheoesophageal Fistula (H-Type, No Atresia)

✅ **Key Clue:**

16. **Delayed diagnosis** (presents with aspiration, recurrent pneumonia)

✅ **Imaging Findings:**

17. **Fluoroscopic Esophagogram (Gold Standard)**
    1. Small contrast-filled fistulous connection to trachea
    2. Needs careful dynamic imaging (may require lateral or oblique view)

✅ **Management:**

18. Surgical repair

---

3. Vascular Anomalies (Esophageal Compression)

19. **Double Aortic Arch** → Posterior indentation (bilateral)
20. **Aberrant Right Subclavian Artery (ARSA)** → Posterior **left-sided** indentation (dysphagia lusoria)
21. **Pulmonary Sling** → Anterior indentation

✅ **Imaging Findings:**

22. **Barium Swallow:** Indentation of esophagus
23. **CT Angiography:** Confirms vascular anatomy

✅ **Management:**

24. Surgical correction if symptomatic

Imaging Approach for Esophageal Obstruction in a Neonate

1️⃣ **CXR with NG Tube Placement** → First-line (confirm atresia vs. distal TEF)

2️⃣ **Fluoroscopic Esophagogram** → For suspected TEF, webs, vascular rings

3️⃣ **CT Angiography** → If vascular anomaly is suspected

Would you like a quick comparison table of esophageal atresia types?

Category: Vascular and Interventional

Best Case – Case 236 (Clarity: 4.5, Trust: 4.5, Differential Usefulness: 4.6, Diagnostic Usefulness: 4.6)

Case 236

Certainly! Here's a **concise, exam-oriented radiological summary** for:

Speciality: Vascular and Interventional Radiology

Topic: Popliteal Artery Occlusion

Definition:

Popliteal artery occlusion is **blockage of the popliteal artery**, typically due to **atherosclerosis**, **embolism**, **thrombosis**, **trauma**, or **entrapment syndrome**, leading to **distal limb ischemia**.

Etiologies (Exam Focus):

1. **Atherosclerosis** (most common in elderly, diabetics, smokers).
2. **Acute thrombosis/embolism** (cardiac source, atrial fibrillation).
3. **Popliteal artery entrapment syndrome (PAES)** (young athletic males).
4. **Trauma (fracture/dislocation)** → vessel injury or dissection.
5. **Aneurysm with thrombosis**.

Clinical Clues:

1. Claudication, cold limb, pain, paresthesia, pallor, pulselessness.
2. Acute limb ischemia vs chronic symptoms (claudication, rest pain, ulcers).

Imaging Modalities & Key Findings:

1. Doppler Ultrasound (First-line)

1. **Absent or monophasic flow** in the popliteal artery.
2. **Turbulent flow** proximal to occlusion.
3. May visualize **echogenic thrombus or plaque**.
4. Good for **dynamic maneuvers in PAES**.

2. CT Angiography (CTA) – Modality of Choice

1. **Abrupt or tapering cutoff** of arterial contrast in occlusion.
2. **Wall calcifications** in atherosclerosis.
3. **Filling defects** in embolism or thrombosis.
4. Assessment of **runoff vessels** (anterior tibial, posterior tibial, peroneal).
5. Identify **entrapment-related compression** or aneurysm.

3. MR Angiography (MRA)

1. Alternative to CTA, especially in **young or renal-compromised patients**.
2. **Dynamic MRA** helpful for suspected entrapment.

4. Digital Subtraction Angiography (DSA) – Gold Standard

1. Precise localization of occlusion.
2. Useful for **intervention planning**.
3. **Collateral circulation assessment**.

Differential Diagnosis:

Management Based on Imaging:

1. **Acute occlusion** → **Thrombectomy**, **thrombolysis**, **stenting**, or **bypass surgery**.
2. **Chronic occlusion** → **Endovascular stenting** or **femoropopliteal bypass**.
3. **PAES** → **Surgical decompression**.
4. **Aneurysm** → **Exclusion via stent graft or surgery**.

Key Exam Pearls:

✅ **CTA is first-line for anatomic detail and intervention planning.**
✅ **Always evaluate runoff vessels and collaterals.**
✅ **PAES = young male, dynamic compression – do functional imaging.**
✅ **Acute vs chronic occlusion differentiation critical for management.**
✅ **Look for associated aneurysm, trauma, or entrapment signs.**

Let me know if you'd like a quick reference table or schematic diagram for quick recall!

Worst Case – Case 239 (Clarity: 4.0, Trust: 3.9, Differential Usefulness: 3.9, Diagnostic Usefulness: 3.4)

Case 239

Certainly! Here's an **exam-focused, concise radiological summary** for:

Speciality: Vascular and Interventional Radiology

Topic: Hypervascular Renal Mass

Definition:

A **hypervascular renal mass** is a lesion with **prominent arterial enhancement** on contrast imaging. Differentiation between **benign and malignant causes** is critical for diagnosis and management.

Primary Differential Diagnoses (Exam-Oriented Table):

Imaging Modality & Enhancement Pattern:

Key Differentiation Clues:

1. **Fat content → AML**
2. **Central scar → Oncocytoma** (but not specific)
3. **Vascular invasion → Suggestive of RCC**
4. **Multiple hypervascular lesions → Consider metastases**

Interventional Relevance:

1. **Pre-op embolization** (especially for large RCCs or AMLs with hemorrhage risk).
2. **Biopsy** considered when imaging is inconclusive (e.g., fat-poor AML vs RCC).
3. **Nephron-sparing ablation** in selected cases (radiofrequency or cryoablation).

Exam Pearls:

✅ Most **RCCs are hypervascular** – especially **clear cell subtype**.
✅ **AML is the most common benign hypervascular renal mass** – look for fat.
✅ **Oncocytoma vs RCC** differentiation is imaging-challenging → biopsy/surgical removal often needed.
✅ **Always assess for venous invasion and metastases**.

Let me know if you'd like a **comparison chart between hypovascular vs hypervascular renal masses**—useful for viva!

Category: Breast

Best Case – Case 293 (Clarity: 4.7, Trust: 4.4, Differential Usefulness: 4.7, Diagnostic Usefulness: 4.4)

Case 293

Well-Circumscribed Breast Cancer – Exam-Oriented Radiological Summary

Common Well-Circumscribed Malignant Breast Lesions

1. **Mucinous (Colloid) Carcinoma**
    1. **Mammography**: Round/oval, well-defined, low-density mass, may have calcifications.
    2. **Ultrasound**: Hypoechoic or isoechoic, posterior enhancement, internal cystic areas.
    3. **MRI**: T2 hyperintense (mucin), progressive enhancement without washout.
2. **Medullary Carcinoma**
    1. **Mammography**: Round, well-circumscribed, high-density mass, no calcifications.
    2. **Ultrasound**: Hypoechoic, posterior enhancement, possible internal vascularity.
    3. **MRI**: T2 hyperintense, rapid enhancement, may mimic benign lesions.
3. **Papillary Carcinoma (Encapsulated or Intracystic)**
    1. **Mammography**: Round/oval, well-circumscribed, may have coarse calcifications.
    2. **Ultrasound**: Solid-cystic lesion, internal vascularity, possible intracystic nodule.
    3. **MRI**: Progressive enhancement, intracystic component with restricted diffusion.
4. **Metaplastic Carcinoma**
    1. **Mammography**: Round, well-defined, high-density mass, no calcifications.
    2. **Ultrasound**: Complex solid-cystic mass, posterior enhancement, possible shadowing.
    3. **MRI**: T2 hyperintense (necrotic areas), irregular progressive enhancement.
5. **Secretory Carcinoma (Juvenile Carcinoma)**
    1. **Mammography**: Well-defined, round mass, may have calcifications.
    2. **Ultrasound**: Hypoechoic or mixed echogenicity, well-circumscribed, posterior enhancement.
    3. **MRI**: Progressive enhancement, heterogeneous internal signal.

Imaging Approach

1. **Mammography**: Assess shape, margins, density, and presence of calcifications.
2. **Ultrasound**: Evaluate internal composition, vascularity, and posterior features.
3. **MRI**: Useful for lesion characterization (T2 hyperintensity, enhancement pattern).
4. **Biopsy**: Essential for histopathological confirmation.

Key Exam Pearls

1. **Well-circumscribed breast mass ≠ benign; certain breast cancers appear circumscribed.**

2. **Mucinous carcinoma → T2 bright with gradual enhancement.**
3. **Papillary carcinoma → Solid-cystic with internal vascularity.**
4. **Medullary carcinoma → Soft, round mass with posterior enhancement.**
5. **If a well-circumscribed lesion lacks classic benign features, biopsy is required.**

Would you like more details on a specific subtype?

Worst Case – Case 291 (Clarity: 2.4, Trust: 2.3, Differential Usefulness: 2.1, Diagnostic Usefulness: 2.2)

Case 291

Bilateral Skin Thickening – Exam-Oriented Radiological Summary

Differential Diagnosis

1. **Vascular Causes**
    1. **Chronic Venous Insufficiency (CVI)** – Lower limb skin thickening with subcutaneous edema, venous dilation, stasis dermatitis, hemosiderin deposition.
    2. **Lymphedema** – Skin thickening with "honeycomb" pattern of subcutaneous fat, absent compressibility on ultrasound.
    3. **Superior Vena Cava (SVC) Syndrome** – Upper body skin thickening, facial swelling, venous collaterals on CT/MRI.
    4. **Post-radiation Changes** – Fibrotic skin thickening, subcutaneous atrophy, reduced vascularity, often asymmetric.
2. **Infectious/Inflammatory Causes**
    1. **Cellulitis** – Ill-defined subcutaneous edema, skin thickening, possible abscess formation.
    2. **Erysipelas** – Superficial skin thickening with sharply demarcated margins.
    3. **Panniculitis (e.g., Erythema Nodosum)** – Septal/subcutaneous fat stranding, skin thickening, no abscess.
    4. **Systemic Lupus Erythematosus (SLE) / Dermatomyositis** – Subcutaneous inflammation with calcifications (lupus panniculitis, calcinosis cutis).
3. **Neoplastic Causes**
    1. **Inflammatory Breast Carcinoma** – Diffuse skin thickening, "peau d’orange" appearance, dermal lymphatic invasion (best seen on mammography/MRI).
    2. **Lymphoma** – Diffuse or nodular skin thickening, soft tissue masses, possible systemic lymphadenopathy.
    3. **Metastases (Carcinomatous Lymphangitis)** – Reticulonodular thickening, diffuse enhancement on CT/MRI.
4. **Other Causes**

1. **Nephrotic Syndrome / Cardiac Failure** – Generalized anasarca, subcutaneous fluid retention.
2. **Hypoalbuminemia** – Diffuse skin thickening from subcutaneous edema.

Imaging Approach

1. **Ultrasound**: Assess skin thickening, subcutaneous edema, vascular patency, rule out abscess.
2. **CT/MRI**: Evaluate for underlying neoplasm, lymphatic/vascular obstruction, extent of skin involvement.
3. **Mammography** (if breast involvement suspected): Evaluate for inflammatory breast carcinoma.
4. **Venous Doppler**: Rule out deep venous thrombosis (DVT) if lower limb involvement.

Key Exam Pearls

1. **Honeycomb pattern in subcutaneous fat → Lymphedema.**
2. **Diffuse non-compressible subcutaneous edema + venous dilation → Chronic venous insufficiency.**
3. **Skin thickening + lung cancer history → Carcinomatous lymphangitis.**
4. **Breast skin thickening + peau d’orange appearance → Inflammatory breast carcinoma.**

Would you like additional details on a specific etiology?
