# Supplementary material for: Large Language Model-Generated Differential Diagnoses in Radiology Education: Comparison with a Standard Casebook
Source: Diagnostics (Basel). 2026 Jun 27;16(13):2009. doi: 10.3390/diagnostics16132009 (PMC13360087; doi:10.3390/diagnostics16132009)
Supplement: Supplementary file 1 [file diagnostics-16-02009-s001.zip › Supplemental Material File S2-TRIPOD.pdf]

Table 2 TRIPOD-LLM checklist

From: The TRIPOD-LLM reporting guideline for studies using large language models

Section	Item	Description	Research design	LLM task	Reported on Section
Title	1	Identify the study as developing, fine-tuning and/or evaluating the performance of an LLM, specifying the task, the target population and the outcome to be predicted.	All	All	Title
Abstract	2	See TRIPOD-LLM for abstracts.	All	All	Abstract
Introduction					
Background	3a	Explain the healthcare context/use case (for example, administrative, diagnostic, therapeutic and clinical workflow) and rationale for developing or evaluating the LLM, including references to existing approaches and models.	All	All	Intro
	3b	Describe the target population and the intended use of the LLM in the context of the care pathway, including its intended users in current gold standard practices (for example, healthcare professionals, patients, public or administrators).	E<br>H	All	Intro
Objectives	4	Specify the study objectives, including whether the study describes the initial development, fine-tuning or validation of an LLM (or multiple stages).	All	All	Intro
Methods					
Data	5a	Describe the sources of data separately for the training, tuning and/or evaluation datasets and the rationale for using these data (for example, web corpora, clinical research/trial data, EHR data or unknown).	All	All	Method
	5b	Describe the relevant data points and provide a quantitative and qualitative description of their distribution and other relevant descriptors of the dataset (for	All	All	Method

Section	Item	Description	Research design	LLM task	Reported on Section
		example, source, languages and countries of origin).			
	5c	Specifically state the date of the oldest and newest item of text used in the development process (training, fine-tuning and reward modeling) and the evaluation datasets.	All	All	Method (Partially)
	5d	Describe any data preprocessing and quality checking, including whether this was similar across text corpora, institutions and relevant sociodemographic groups.	All	All	Method
	5e	Describe how missing and imbalanced data were handled and provide reasons for omitting any data.	All	All	Method
Analytical methods	6a	Report the LLM name, version and last date of training.	All	All	Method/Zenodo
	6b	Report details of the LLM development process, such as LLM architecture, training, fine-tuning procedures and alignment strategy (for example, reinforcement learning and direct preference optimization) and alignment goals (for example, helpfulness, honesty and harmlessness).	M<br>D	All	Method/Zenodo
	6c	Report details of how the text was generated using the LLM, including any prompt engineering (including consistency of outputs), and inference settings (for example, seed, temperature, max token length and penalties), as relevant.	M<br>D<br>E	All	Method/Zenodo
	6d	Specify the initial and postprocessed output of the LLM (for example, probabilities, classification and unstructured text).	All	All	Method/Zenodo
	6e	Provide details and rationale for any classification and, if applicable, how the probabilities were determined and thresholds identified.	All	C<br>OF	Method

Section	Item	Description	Research design	LLM task	Reported on Section
				QA	Method
	7a	Include metrics that capture the quality of generative outputs, such as consistency, relevance, accuracy and presence/type of errors compared to gold standards.	All	IR<br>DG<br>SS<br>MT	
	7b	Report the outcome metrics' relevance to the downstream task at deployment time and, where applicable, the correlation of metric to human evaluation of the text for the intended use.	E<br>H	All	Method
LLM output	7c	Clearly define the outcome, how the LLM predictions were calculated (for example, formula, code, object and API), the date of inference for closed-source LLMs and evaluation metrics.	E<br>H	All	Method
	7d	If outcome assessment requires subjective interpretation, describe the qualifications of the assessors, any instructions provided, relevant information on demographics of the assessors and interassessor agreement.	All	All	Method
	7e	Specify how performance was compared to other LLMs, humans and other benchmarks or standards.	All	All	Method
	8a	If annotation was done, report how the text was labeled, including providing specific annotation guidelines with examples.	All	All	N/A
Annotation	8b	If annotation was done, report how many annotators labeled the dataset(s), including the proportion of data in each dataset that was annotated by more than one annotator, and the interannotator agreement.	All	All	N/A
	8c	If annotation was done, provide information on the background and experience of the annotators or the	All	All	N/A

Section	Item	Description	Research design	LLM task	Reported on Section
		characteristics of any models involved in labeling.			
Prompting	9a	If research involved prompting LLMs, provide details on the processes used during prompt design, curation and selection.	All	All	Method
	9b	If research involved prompting LLMs, report what data were used to develop the prompts.	All	All	Method
Summarization	10	Describe any preprocessing of the data before summarization.	All	SS	Method
Instruction tuning/alignment	11	If instruction tuning/alignment strategies were used, what were the instructions, data and interface used for evaluation, and what were the characteristics of the populations doing the evaluation?	M<br>D	All	N/A
Compute	12	Report compute, or proxies thereof (for example, time on what and how many machines, cost on what and how many machines, inference time, floating-point operations per second), required to carry out methods.	M<br>D<br>E	All	N/A
Ethical approval	13	Name the institutional research board or ethics committee that approved the study and describe the participant-informed consent or the ethics committee waiver of informed consent.	All	All	N/A
Open science	14a	Give the source of funding and the role of the funders for the present study.	All	All	Statements
	14b	Declare any conflicts of interest and financial disclosures for all authors.	All	All	Statements
	14c	Indicate where the study protocol can be accessed or state that a protocol was not prepared.	H	All	N/A
	14d	Provide registration information for the study, including register name and registration number, or state that the study was not registered.	H	All	N/A
	14e	Provide details of the availability of the study data.	All	All	Statements

Section	Item	Description	Research design	LLM task	Reported on Section
	14f	Provide details of the availability of the code to reproduce the study results.	All	All	N/A
Public involvement	15	Provide details of any patient and public involvement during the design, conduct, reporting, interpretation or dissemination of the study or state no involvement.	H	All	N/A
Results					
Participants	16a	When using patient/EHR data, describe the flow of text/EHR/patient data through the study, including the number of documents/questions/participants with and without the outcome/label and follow-up time as applicable.	E<br>H	All	N/A
	16b	When using patient/EHR data, report the characteristics overall and for each data source or setting and development/evaluation splits, including the key dates, key characteristics and sample size.	E<br>H	All	N/A
	16c	For LLM evaluation that includes clinical outcomes, show a comparison of the distribution of important clinical variables that may be associated with the outcome between development and evaluation data, if available.	E<br>H	All	N/A
	16d	When using patient/EHR data, specify the number of participants and outcome events in each analysis (for example, for LLM development, hyperparameter tuning and LLM evaluation).	E<br>H	All	N/A
Performance	17	Report LLM performance according to prespecified metrics (see item 7a) and/or human evaluation (see item 7d).	All	All	Results
LLM updating	18	If applicable, report the results from any LLM updating, including the updated LLM and subsequent performance.	All	All	N/A
Discussion					

Section	Item	Description	Research design	LLM task	Reported on Section
Interpretation	19a	Give an overall interpretation of the main results, including issues of fairness in the context of the objectives and previous studies.	All	All	Discussion
Limitations	19b	Discuss any limitations of the study and their effects on any biases, statistical uncertainty and generalizability.	All	All	Discussion
	19c	Describe any known challenges in using data for the specified task and domain context with reference to representation, missingness, harmonization and bias.	E<br>H	All	Discussion
	19d	Define the intended use for the implementation under evaluation, including the intended input, end-user and level of autonomy/human oversight.	E<br>H	All	Discussion
Usability of the LLM in context	19e	If applicable, describe how poor quality or unavailable input data should be assessed and handled when implementing the LLM; that is, what is the usability of the LLM in the context of current clinical care.	E<br>H	All	Discussion
	19f	If applicable, specify whether users will be required to interact in the handling of the input data or use of the LLM, and what level of expertise is required of users.	E<br>H	All	Discussion
	19g	Discuss any next steps for future research, with a specific view of the applicability and generalizability of the LLM.	All	All	Discussion

1. For studies using existing LLMs, users should include reference(s) to reportable information if provided by the original developers or state that this information is not available.
2. M, LLM methods; D, de novo LLM development; E, LLM evaluation; H, LLM evaluation in healthcare settings; C, classification; OF, outcome forecasting; QA, long-form question answering; IR, information retrieval; DG, document generation; SS, summarization and simplification; MT, machine translation; API, application programming interface.
